# HMGCS2-dependent β-OHB/H3K9bhb ameliorates synaptic plasticity and cognition in Alzheimer’s disease

**DOI:** 10.1038/s12276-026-01664-9

**Published:** 2026-03-06

**Authors:** Haitao Yu, Fangzhou Wang, Jia-qi Yuan, Jia Chen, Ke-yu Zhang, Dongdong Jia, Juan Gong, Yuming Mao, Shuguang Bi, Yu-qi Zhang, Zi-chong Lan, Hao-yan Yu, Gao-shang Chai

**Affiliations:** 1 Department of Fundamental Medicine, Wuxi School of Medicine, Jiangnan University; MOE Medical Basic Research Innovation Center for Gut Microbiota and Chronic Diseases, School of Medicine, Jiangnan University, Wuxi, People’s Republic of China; 2 Affiliated Hospital of Jiangnan University, Wuxi, People’s Republic of China; 3 The Affiliated Mental Health Center of Jiangnan University, Wuxi Central Rehabilitation Hospital, Wuxi, People’s Republic of China

**Keywords:** Histone analysis, Long-term potentiation

## Abstract

Ketogenic diet (KD) can significantly ameliorate cognition in Alzheimer’s disease (AD), but the specific mechanism is not clear. Histone3-lysine9-β-hydroxybutyrylation (H3k9bhb), a novel histone modification mark induced by ketogenesis-generated β-hydroxybutyrate (β-OHB), may be involved in the prevention and treatment of AD. Here we report that β-OHB and H3K9bhb were reduced in the hippocampus of triple transgenic AD male mice (3xTg-AD) mice. Reduced H3K9bhb levels were also observed in patients with AD. The 3xTg-AD mice exhibited a low enrichment of H3K9bhb on the promoters of NMDA receptor subunits and Syn1 and axon-related genes together with impaired synaptic plasticity, all of which were rescued by 3-hydroxy-3-methylglutaryl-CoA synthase 2 (HMGCS2, a rate-limiting enzyme of β-OHB synthesis) upregulation. Moreover, β-OHB replenishment enhanced H3K9bhb in 3xTg-AD mice, leading to an increase of NMDA receptor subunits and Syn1 and cognitive function in an HMGCS2-dependent manner. Thus, HMGCS2 is a key molecular switch of cognitive impairment, and targeting HMGCS2 or β-OHB replenishment appropriately may serve as a novel therapeutic strategy for AD treatment.

## HMGCS2/β-OHB-mediated H3K9bhb ameliorates AD cognition

Alzheimer’s disease (AD) is a common brain disorder that affects memory and thinking. Although ketogenic diets benefit AD, the specific mechanism remained unclear. Yu and colleagues identified a key metabolic enzyme that restores cognitive function in AD by regulating a specific epigenetic modification required for healthy brain connections. The researchers found that AD brains lack HMGCS2, the enzyme producing β-hydroxybutyrate (β-OHB). This molecule is crucial for creating H3K9bhb, an epigenetic marker that activates genes essential for synaptic communication. In AD mice, increasing HMGCS2 or supplementing β-OHB restored H3K9bhb levels, reactivated synaptic genes such as *NMDARs* and *Syn1* and significantly improved memory. Thus, HMGCS2 acts as a key molecular switch, suggesting that targeting it or supplementing β-OHB offers a novel therapeutic strategy for AD.

This summary was initially drafted using artificial intelligence, then revised and fact-checked by the author.

## Introduction

Alzheimer’s disease (AD) is the most common neurodegenerative disease, and its main clinical manifestations are cognitive impairment and progressive decline of memory ability^1^. The main pathology of AD includes amyloid beta (Aβ) plaque deposition and neurofibrillary tangles (NFTs) formed by hyperphosphorylated tau^2,3^. Current research indicates that the typical AD pathologies of Aβ and NFTs appear 15–20 years before cognitive impairment occurs. Aβ and NFTs can not only induce inflammation in glial cells^4^ and mitochondrial dysfunction^5^ in the brain but also promote axonal and synaptic damage and functional degradation^6–9^. Synapses are the functional executive units of learning and memory, and their damage is crucial in mediating the progression of AD from preclinical stage to mild cognitive impairment, leading to dementia^10,11^. Therefore, searching for potential methods and related mechanisms to reverse synaptic dysfunction is of great significance for AD treatment.

As a typical way of epigenetic regulation, histone modification plays an important role in the regulation of cognitive function in AD^12^ via affecting chromatin remodeling and participating in the regulation of many neuronal functions, from synaptic plasticity to learning and memory^13^. Histone protein β-hydroxybutylation modification (Kbhb) is a newly discovered lysine modification that activates chromatin, thereby promoting gene expression^14^. Research had shown that histone Kbhb was closely related to the pathogenesis of cardiovascular disease, kidney disease, tumors and mental illness, suggesting that it may co-mediate pathological and physiological changes in diseases similar to other histone modifications^15^. At present, mass spectrometry analysis has identified multiple lysine sites that can generate histone Kbhb, including H3K4, H3K9, H3K18, H4K8, H4K12, H4K16 and so on^14^. The current research mainly focuses on histone3-lysine9-β-hydroxybutyrylation (H3k9bhb), which was closely related to amino acid catabolism, circadian rhythm, redox balance, PPAR signaling pathway and oxidative phosphorylation^14^. Mouse experiments have shown that H3K9bhb may directly affect brain cell function by reshaping chromatin structure and transcriptional responses^16^. The above studies indicated that histone Kbhb was closely related to the pathogenesis of various diseases, but its specific role and mechanism in neurodegenerative diseases, especially in AD, has not been reported yet.

β-hydroxybutyrate (β-OHB), as the main component of ketones, is produced by fatty acid β-oxidation under fasting, exercise, calorie restriction, ketogenic diet and other conditions, accompanied by an increase in intracellular histone Kbhb modification, which in turn regulates gene expression or levels of other types of histone modification^17,18^. Specifically, β-OHB was catalyzed by 3-hydroxy-3-methylglutaryl-CoA synthase 2 (HMGCS2) to condense acetoacetyl-CoA (AcAc-CoA) and acetyl-CoA to produce 3-hydroxy-3-methylglutaryl-CoA (HMG-CoA), whereas HMG-CoA lyase (HMGCL) cleaves HMG-CoA into acetyl-CoA and acetyl acetate (AcAc), of which, the latter was catalyzed by hydroxybutyrate dehydrogenase 1 (BDH1)^19^. Among them, HMGCS2 and BDH1, as key enzymes in ketone metabolism, were closely related to the modification of histone Kbhb. The deficiency of HMGCS2 in the intestine could impair the aggregation of H3K9bhb and affect the metabolic gene program related to H3K9bhb^17^, whereas inhibiting BDH1 could lead to β-OHB aggregation, increase H3K9bhb and promote liver cancer cell proliferation^20^. Research had shown that β-OHB was significantly reduced in the blood and brain tissues of patients with AD^21^, and ketogenic diet could alleviate cognitive impairment in patients with AD^22^. However, the specific mechanism of β-OHB generation disorders and their key role in regulating learning and memory abilities are still unclear. 

In this study, we focused on the key changes of H3K9bhb in AD brain tissue, revealing that HMGCS2 was a key molecule that promotes the production of β-OHB and then improves AD cognitive function by increasing H3K9bhb to regulate synaptic plasticity. The research results will reveal new epigenetic regulatory mechanisms in AD, providing new ideas and targets for the development of prevention and control strategies.

## Materials and methods

### Experimental animals and human post-mortem tissues

Triple transgenic AD male mice (3xTg-AD) (stock no.: 34830, 129S4.CgTg (APPSwe, tauP301L) 1LfaPsen1tm1Mpm/Mmjax) mice and wild-type (WT) mice were a gift from Prof. Xifei Yang (Shenzhen Center for Disease Control and Prevention). The experimentation was authorized by the Animal Ethics Committee of Jiangnan University (JN. no. 20240229m0450930[067]). The area in which they were fed maintained a temperature range of 22–26 °C, whereas the relative humidity was kept between 50% and 60%. Adequate food and water were ensured, with 12-h light–dark alternating. It is important to note that all experiments conducted with these mice adhere to the ethical considerations and animals’ well-being. For the 6-month-old 3xTg-AD mice, β-OHB (Sigma, H6501) was administered for 3 months at a dose of 3 g/kg and a final concentration of 0.01875 g/ml in water^21^.

Frozen postmortem brain tissues from patients with AD and normal control subjects were acquired from Guizhou Medical University, and further information is shown in Supplementary Table 1. Human brain tissue samples were approved by the ethics committee of Guizhou Medical University and ethics committee of Jiangnan University (JNU202403RB095).

### MWM test

First, a series of learning and training experiments were conducted over six consecutive days. Mice were placed in the water maze from four quadrants, and the time it took them to find the underwater platform was recorded. Memory detection experiments were performed on the second day after the learning and training phase. Several measures were recorded during this experiment, including the time it took for the mice to first cross the platform, the number of crossings made within 60 s and the distance and time taken to reach the target quadrant. These measures enabled an assessment of memory retention and spatial learning abilities in the mice.

### NOR test

Mice were placed in a 60 cm × 60 cm × 50 cm white box to acclimate for 1 day. After the formal experiment, an identical cylinder was placed at each end of the box, and the mice were allowed to explore freely in the middle for 10 min. The next day, one of the cylinders was replaced with a cone, and the mice were again allowed to explore freely for 10 min. During the experiment, the time spent in the target object area and the number of times the mice explored the target object were recorded.

### Fear condition test

The experimental chamber (230 mm × 230 mm × 300 mm) had a stimulated electric grid at the bottom and was housed in a soundproof chamber (600 mm × 600 mm × 600 mm). On the first day after the experiment, short-term memory was tested, and on the seventh day after the experiment, long-term memory was tested, and the freezing time of each mouse in 3 min was recorded.

### Golgi staining

The mice were anesthetized with 20% ulatane; the fresh brain was removed on ice, and external blood stains were rinsed with PBS (BL302A 1× PBS, Biosharp). The staining was performed using a Rapid GolgiStain kit (PK401, FD Neuro Technologies PK40). Images were taken under the light field (Axio Imager Z2 Carl Zeiss).

### RNA sequencing analysis

Transcriptomics comprehensively characterized the hippocampus gene expression of 3xTg-AD mice or the 3xTg-AD+β-OHB group, including RNA purification, reverse transcription, library construction and sequencing. To gain insights into the biological functions and pathways associated with the differentially expressed genes, the Kyoto Encyclopedia of Genes and Genomes (KEGG) and the Web-Based Gene Set Analysis Toolkit (http://www.webgestalt.org) were utilized for performing these analyses.

### CUT&Tag sequencing and data quality control

Chromatin immunoprecipitation sequencing (ChIP) assays were performed by CUT&Tag 4.0 High-Sensitivity Kit (N259-YH01, Novoprotein). In brief, the hippocampus of mice was processed into cell suspension in the same way as ChIP. All brain cells were collected and bound to 10 mM concanavalin A-coated beads for 10 min at room temperature. Subsequently, cells were resuspended in primary antibody buffer and sequentially incubated with primary and secondary antibodies to H3K9bhb (6 μg, Proteintech, PTM-1250RM) overnight at 4 °C with gentle rotation. Next, the samples were incubated with pA-Tn5 transposase in ChiTag buffer. After the transposon was activated by Mg^2+^ at 37 °C for 1 h, the fragmentation of DNA was completed. The fragmentation of DNA was terminated with 5 μl stop buffer and 1 μl proteinase K for 2 h at 55 °C, and the library was constructed after DNA isolation, amplification and purification were performed.

Mouse hippocampal samples were subjected to CUT&Tag library preparation, and the library integrity (200–1000 bp) was confirmed by 1% agarose gel electrophoresis. Libraries were sequenced on the Illumina NovaSeq Xplus platform with paired-end 50–150-bp reads, yielding a total of 47.56 G of raw data. Raw reads were quality-filtered using Fastp (v0.22.0) to remove adapters, low-quality bases (*Q* <20), high N content (>30%) and short reads (<50 bp). Clean reads were aligned to the mouse reference genome using Bowtie2 (v2.4.1, default parameters), achieving a >95% mapping rate, Q20 >90%, Q30 >85% and >10,000 called peaks, indicating high-quality data with sufficient biological information. BAM files were used for peak calling with MACS3 (v3.0.0a7). Overlapping peaks were merged using bedtools (v2.30.0, default parameters), and read counts in merged peaks were quantified with FeatureCounts (v2.0.3). Differential peak analysis was performed with DESeq2 (v1.38.3, size factor normalization) according to the presence of biological replicates, using |log_2_ fold change| >1 and *P* < 0.05 as significance thresholds. The signal visualization was generated with Deeptools (v3.5.1), peak annotation with ChIPseeker (v1.34.1), functional enrichment analysis with topGO (v2.50.0) and clusterProfiler (v4.6.0) and motif analysis with HOMER (v4.11).

### ChIP–qPCR

Chromatin immunoprecipitation (ChIP) assays were performed by ChIP assay kit (P2087, Beyotime). In brief, the hippocampus of mice was cross-linked with 1% formaldehyde for 10 min in cold PBS. Then, the crosslinking was quenched by adding glycine solution (10×) and washed twice with cold phenylmethylsulfonyl fluoride (PMSF) (1 mM) containing PBS. The SDS lysis buffer containing PMSF (1 mM) was used to homogenize the tissue. The chromatin fragments (∼200–1000 bp in length) were obtained by sonication (Sonics VCX 150) at 30% power with 12 rounds of 30 s pulse (30 s pause between pulses) at 4 °C, whereas the samples were immersed in an ice-water bath. Then, we could obtain sheared chromatin from supernatant after centrifugation at 12,000*g* for 5 min at 4 °C. The chromatin was incubated with antibody against H3K9bhb (6 μg, Proteintech, PTM-1250RM) overnight at 4 °C with gentle rotation after diluted with ChIP dilution buffer containing PMSF (1 mM); mouse IgG was used as the negative control. Immunocomplexes were precipitated by Protein A + G Agarose/Salmon Sperm DNA and washed sequentially with low-salt, high-salt, LiCl and TE buffer. The protein–DNA cross-linking was reversed by incubation with 4 µl 5M-NaCl at 65 °C for 4 h after eluted with elution buffer (1% SDS, 0.1 M NaHCO3), and DNA fragments were purified with PCR clean up kit (D0033, Beyotime). The 0–2000-bp fragments around the transcription start sites region of related genes were amplified with SYBR-Green Real-Time PCR Kit and the Light Cycler 480 Real-Time PCR detection system. All the PCR primers used are listed in Table 1. Ct values are obtained from both ChIP and input samples. The dilution factor of the input sample was taken into account. Results are expressed as the percentage of ChIP signal relative to the Input signal. Subsequently, the data are normalized and presented relative to the WT control for comparative analysis.

**Table 1:** PCR primers used in the present study.

Gene	Forward primer (5′ → 3′)	Reverse primer (5′ → 3′)
*Cxcl2*	GCTCTGCATCAGTGACGGTA	TAATTTCGGGTCAATGCACA
*Ppp3ca*	GGGACATCCATGGACAATTC	AAGGCCCACAAATACAGCAC
*Plxnb3*	ATATGCTGAGCGTGCCTTCT	TGCTGTTGAGCAAATTGGAG
*Sema6d*	AGACACGGGTCAGGTACAGG	AAGCATGGTGATCCTTGTCC
*Robo3*	AAGGATTCCGTGTGTCTTGG	GGGCAGTCCTCTTAGCACAG
*Cxcl2 ChIP*	CCTCTAGCCCTCCAAGATTCA	AAAGTGGTCATTGGCGCTTT
*Ppp3ca ChIP*	GAGTGTGTGCAGGACTTCG	TTTCTTCAGGGTGCCAGTCA
*Plxnb3 ChIP*	TGAGCTTAGAGGGAGGGTCT	CACCAAGGTACAGAAGGGGA
*Sema6d ChIP*	TAGAACTGTGACTTGGGCGG	TCCAATCTGCATCCCTTCCC
*Robo3 ChIP*	TGTGGGTTCCTAGTGGCAAG	ATTCTGGGTCCTGGAAACGT
*Glun1*	CCTACTCCCAACGACCACTT	AGACGCGCATCATCTCAAAC
*Glun2A*	TTGGGAGCGGGTACATCTTT	CTCCTGCCATGTTGTCGATG
*Glun2B*	GGAGAGGGTGGGAAAATGGA	ACAATGACAAACGGTGCCTC
*Syn1*	CTCAGCAGCACAACATACCC	GGTCTTCCAGTTACCCGACA
*Glun1 ChIP*	TGTGCAGACATGGAACCTC	AGAATCCTGGACCCACACG
*Glun2A ChIP*	ACCTTCCCTTAGCACTGGTG	CACCAGGTTTAGCGGGTTC
*Glun2B ChIP*	AGAGATTGCACGTGAGGAAA	CACACGCCATGCAACAGATT
*Glun2C ChIP*	AGACATACAAGTTCTGGAGGAGA	GACAGCCTCTTTGCCAGATG
*Syn1 promoter*	TCCACAGACAACAGACACAGA	GGACTGGTGCTGTTTGGAAA
*HMGCS1*	TTTGATGCAGCTGTTTGAGG	CCACCTGTAGGTCTGGCATT
*HMGCS2*	GGCTGTCAAAACAGTGCTCA	GCAATGTCACCACAGACCAC
*HMGCL*	CCAAGGAAGTGAGCGTCTTC	TGAGTCCTGGGGTACCTACG
*BDH1*	CGGCTAGTGGCAAAGCTATC	GTTGCAGACATTGAGCTGGA
*ACAT1*	TATTTCCACTCCATGCACCA	ATTGGACATGCTCTCCATCC
*ACAT2*	CTACCACATGGGCATCACAG	TGACAGTTCCTGTCCCATCA
*Shank2*	GAGGAAGAGCGGCAGTTTCT	CACGTCTTCTCGGGGCTATC
*Cacna1c*	TCTTCCACCCCAACGCTTAC	TCCAGCCCTCCATGGTGATA
*Synj1*	GAGCACCGTCAAGAACTCCA	AGTAGGTGCTGCTATGGGGA
*Syt7*	AGGGCTTTCGGAACAGGATG	CCTCTGCACCACTTTCTCGT
*Syt8*	GCCAGGAGATTAGAGTGGGC	GTCGTGCCTTTCTTGGAGGA
*Cttn*	TAGTGGCTTCGGTGGCAAAT	CCTGTCCTCCTGCTCTCTCT
*Lrrc4c*	TGGAACAGTCATGACCCACG	GTTTTGCCGATGGTGCTGTT
*Mycbp2*	CTGGACGTCTCGTATGAGCC	CCAGGCTTCAGTGTAACCGT
*Shank2 (ChIP)*	GGCCAGGGCTTCTCTTCTTT	TATTGGTGGAGGGGAGGGAG
*Cacna1c (ChIP)*	CCCGGATGTACTGAGGATGC	CAGGGTCAGACACAGCAGAG
*Synj1 (ChIP)*	CACAACCTGTTCTTCAGCGC	GTTGGTCTTTCATCCCCGGT
*Syt7 (ChIP)*	CCTGGAGCCTTGTTTTGCAC	AGTGGTATGCAGAAAGGGCC
*Syt8 (ChIP)*	GTCCAAAACCACAGGAACGC	ATGGTGCTATGCTGTGGCTT
*Cttn (ChIP)*	CAAACGCTGACACCATTGCA	TGATCTCCACCTCCCCAGAG
*Lrrc4c (ChIP)*	ATGGATGCAGGACAGTGACG	ATCCAAAATCAGGGGGCTGG
*Mycbp2*	GGCAGCTCTGTGTGGTAGTT	CCACATGCTGGTTACCTGGT

### Reverse transcription and real-time qPCR

Total RNA was extracted from mouse brain tissue by Trizol method and reverse transcribed to produce complementary DNA (R223, Vazyme Biotech). The real-time PCR was performed using 0.8 μl forward and 0.8 μl reverse primers, 1 μl complementary DNA (cDNA), 10 μl SYBR green PCR parent (731360ES50, Yesson Biotechnology) and 7.4 μl diethyl pyrocarbonate water. All the PCR primers used are listed in Table 1.

### Western blotting assays

All the primary antibodies (Table 2) were used to detect histone Kbhb (H3K9bhb, H3K4bhb, H4K16bhb), histone acetylation (H4K12ace, H4K5ace), histone (H3, H4), synaptic plasticity (Glun1, Glun2A, Glun2B, SYN1) and HMGCS2 and HRP-linked secondary antibody (anti-rabbit or anti-mouse IgG conjugated to horseradish peroxidase, 1:5000).

**Table 2:** Antibodies used in western blotting and their properties.

Antibody	Specificity	Type	Dilution for WB or IF	Source	Cataloge number
Kbhb	β-hydroxybutyryllysine	Mono-	1:1000	PTM BIO	PTM-1201RM
H3K9bhb	β-hydroxybutyryl-histone H3 (Lys9)	Mono-	1:1000 (1:100 for IF)	PTM BIO	PTM-1250RM
H3K4bhb	β-hydroxybutyryl-histone H3 (Lys4)	Mono-	1:1000	PTM BIO	PTM-1296
H4K16bhb	β-hydroxybutyryl-histone H4 (Lys16)	Mono-	1:1000	PTM BIO	PTM-1262
Histone H3	Human histone H3	Mono-	1:1000	Abcam	ab176842
Histone H4	Human histone H4	Mono-	1:1000	Abcam	ab177840
H4K12ace	Histone H4 acetylated at lysine 12	Poly-	1:5000	Abcam	ab46983
H4K5ace	Histone H4 acetylated at lysine 5	Mono-	1:10,000	Abcam	ab51997
β-Actin	β-actin	Poly-	1:1000	SAB	21338
SYN1	Synapsin-1	Poly-	1:1000 (1:100 for IF)	SAB	41470
SYN1	Synapsin-1	Poly-	1:1000	Proteintech	20258-1-AP
GluN1	GluN1	Mono-	1:1000	SAB	49488
GluN2A	NMDAR2A	Poly-	1:1000	Proteintech	28525-1-AP
GluN2B	NMDAR2B	Poly-	1:1000	Proteintech	21920-1-AP
PSD95	PSD95	Poly-	1:1000	Proteintech	20665-1-AP
HMGCS2	HMGCS2	Poly-	1:1000 (1:100 for IF)	ABclonal	A14244
NeuN	Human NeuN amino acids 1–100 (N-terminal)	Mono-	1:100 for IF	Abcam	ab104224
Map2	MAP2	Poly-	1:100 for IF	SAB	32723
4G8	β-amyloid, 17-24	Mono-	1:200 for IF	BioLegend	800701
IBA1	IBA1	Mono-	1:100 for IF	1:100 for IF	60190-1-lg
GFAP	GFAP	Mono-	1:100 for IF	1:100 for IF	66827-1-lg
PPARα	PPARα	Mono-	1:3000	Proteintech	66826-1-IG

Mono-, monoclonal; Poly-, polyclonal; WB, western blotting; IF, immunofluorescence.

### Immunofluorescence

Coronal brain sections (12 µm) were stored at −20 °C in cryoprotective solution (30% sucrose, 1% polyvinylpyrrolidone, 5 mM Na₂HPO₄, 20 mM NaH₂PO₄ and 30% ethylene glycol) until use. Sections were washed three times with PBS and permeabilized with 0.5% Triton X-100 (LA1604602, National Medicine) in PBS at room temperature for 30 min, followed by three washes with PBS (5 min each). The nonspecific binding was blocked with 5% BSA in PBS for 60 min at room temperature. The sections were incubated with anti-H3K9bhb, anti-NeuN, anti-MAP2, anti-GFAP, anti-IBA1 and anti-HMGCS2 primary antibody for 24 h at 4 °C (Table 2). The second day, the brain sections were washed with 0.1% TritonX-100 in PBS three times and incubated with donkey-anti-rabbit Alexa Fluor 488 (Jackson ImmunoResearch, 711-545-152) or donkey-anti-mouse Alexa Fluor 594 (Jackson ImmunoResearch, 715-585-150) for 1 h at room temperature. Pictures were visualized by LSM710 (Zeiss Carl LSM 710).

### Immunohistochemistry

According to the instructions of the DAB kit (CW2069S, CWBIO), mouse paraffin-embedded sections were first deparaffinized in xylene and rehydrated through a graded alcohol series. Following the antigen retrieval, the sections were treated with an endogenous peroxidase blocking solution and incubated at room temperature for 10 min. After blocking with goat serum, the slides were incubated with a primary antibody working solution overnight at 4 °C. The next day, a secondary antibody working solution was applied and incubated at room temperature for 10 min, followed by incubation with horseradish peroxidase (HRP)-conjugated streptavidin for another 10 min at room temperature. The color development was performed using the DAB working solution. Subsequently, the stained sections were counterstained, dehydrated, cleared and mounted. Finally, the slides were examined under a Nikon microscope (80i, Nikon) for immunohistochemical analysis.

### β-OHB colorimetric assay kit

β-OHB levels in the hippocampus were measured using a mouse β-OHB colorimetric assay kit (Michigan Cayman chemical Biotech, 700190) according to the manufacturer’s instructions.

### Primary neuron and N2a cell culture and treatment

The murine neuroblastoma Neuro2a cells (N2a) was a gift from Prof. Jian-Zhi Wang (Tongji Medical School). The cells were cultured in Dulbecco’s modified Eagle medium (DMEM) with 5% fetal bovine serum (FBS) and penicillin–streptomycin solution (1×). The culture medium was changed every 2 days.

Primary neurons used in our vitro studies were prepared from C57BL6/J and 3xTg-AD mice pups (1 day old). The cells isolated from the cerebral cortex and hippocampus were plated at a density of 3 × 10^5^ cells/cm^2^ in DMEM with 5% FBS and penicillin–streptomycin solution (1×) for 3–4 h. The cultures were transferred to maintenance medium contained by neurobasal with 2% B27 (50×) and 1% glutamine after the cells were attached to the poly-l-lysine-coated 75 cm^2^ culture flasks or 48-well plates. The culture medium was changed every 2 days.

### Dual-luciferase reporter assay

The sequence of interest was cloned into a dual-luciferase reporter system containing both firefly and *Renilla* luciferases. The resulting plasmid was co-transfected into N2a cells with either a peroxisome proliferator-activated receptor alpha (PPARα)-overexpression plasmid, a PPARα-knockdown plasmid or an empty vector control. After 48 h, the cells were collected and lysed. According to the manufacturer’s protocol for the dual-luciferase reporter assay, the firefly luciferase substrate was added to the lysates, and the relative light units (RLU) were measured using a multimode microplate reader. Subsequently, the *Renilla* luciferase working solution was added, and the RLU was recorded. Firefly luciferase activity was used as the experimental reporter, whereas *Renilla* luciferase served as the internal control. The ratio of RLU (firefly) to RLU (*Renilla*) was calculated to evaluate the transcriptional activation of the target gene.

### Isolation of neurons from adult mouse brains

Adult mice were deeply anesthetized with an isoflurane gas anesthesia machine, and their hearts were infused with precooled normal saline. The whole brain was immediately removed without the cerebellum and washed with pre-cooled PBS. According to the manufacturer’s instructions, enzymatic hydrolysis of brain tissue was carried out using the adult brain isolation kit (130-107-677, Miltenyi Biotec). After removing myelin with the cell debris-removal buffer, the cell precipitate was resuspended in PBS containing 2% BSA, and then, specific cell types were isolated. The method of cell sorting by magnetic beads was adopted. According to the manufacturer’s instructions, mouse neurons were isolated using the adult neuron isolation kit (130-126-603, Miltenyi Biotec)^23^. The sorted cells were subjected to the next ChIP experiment.

### Statistical analysis

Data were analyzed using GraphPad Prism software 9 (GraphPad Software). The statistical analysis was performed by a two-way analysis of variance (ANOVA) followed by Bonferroni’s post hoc test, two-way repeated-measures ANOVA, followed by Bonferroni’s post hoc test or Student’s *t*-tests. The data were expressed as mean ± s.e.m., and *P* values <0.05 were considered to be significant.

## Results

### β-OHB and histone β-hydroxybutyrylation levels were decreased in the brains of 3xTg-AD mice and patients with AD

**Fig. 1:**
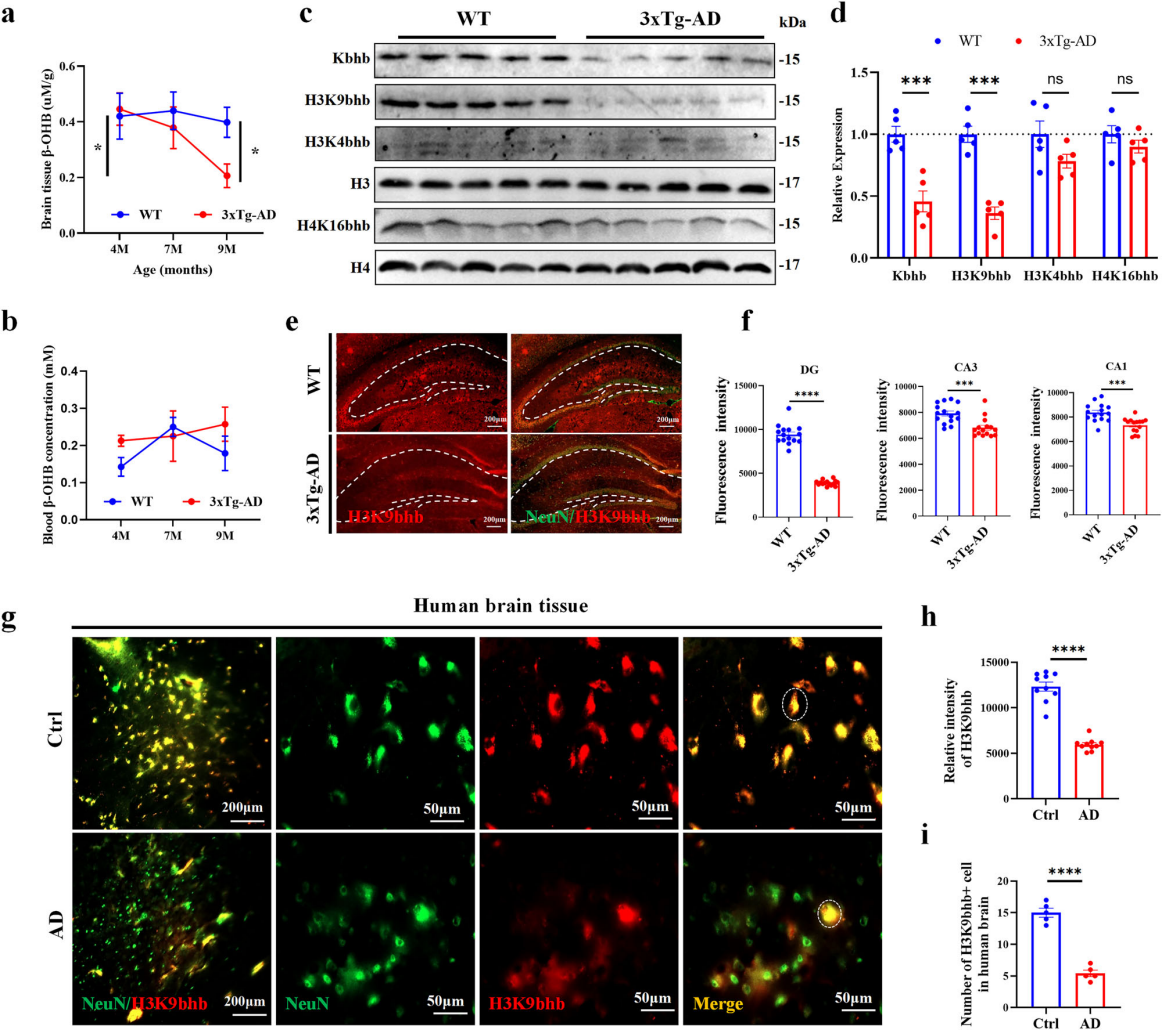
Histone β-hydroxybutyrylation was significantly decreased in brain tissue of 3xTg-AD mice and patients with AD. **a**,**b**, The levels of β-OHB in the hippocampus, *n* = 6 per group (**a**) and blood, *n* = 5 per group (**b**) of WT and 3xTg AD mice at different ages. **c**,**d**, The western blotting analysis of pan- and site-specific hippocampal β-hydroxybutylation (H3K9, H3K4, H4K16) in the hippocampus of 9-month-old WT and 3xTg-AD mice, *n* = 5 per group. **e**,**f**, The immunofluorescence were used to measure the expression level of H3K9bhb in hippocampal neurons of 9-month-old WT and 3xTg-AD mice, *n* = 5 per group, three fields per mice. Scale bar, 200 μm. **g**–**i**, The immunofluorescence was used to measure the H3K9bhb level in the brain of patients with AD (**g**), *n* = 4 per group, ten fields per group (**h**). The number of H3K9bhb in human brain (**i**). Scale bar, 50 μm. Data are shown as mean ± s.e.m. Unpaired *t*-test for **d**, **f**, **h** and **i**. Two-way ANOVA followed by Bonferroni’s post hoc test for **a** and **b**. ^*^P < 0.05, ^**^P < 0.01, ^***^P < 0.001, ^****^P < 0.0001; ns, not significant.

The pathological development and neurodegeneration of AD can be driven by metabolic dysfunction in the brain^24,25^. β-OHB, the main component of ketone bodies, can improve synaptic plasticity and learning and memory function in AD mice by reducing the deposition of Aβ plaque deposition and the level of phosphorylated tau, inhibiting the excessive activation of microglia and enhancing mitochondrial function^21,26–28^. We first examined the level of β-OHB in the hippocampus of 3xTg-AD mice. The level of β-OHB in the hippocampus of 9-month-old 3xTg-AD mice was significantly reduced compared with WT mice (Fig. 1a), which has been found to be consistent with previous clinical reports that the level of β-OHB in the brain tissue of patients with AD was significantly lower than that of healthy controls^21^. Interestingly, there was no significant change in blood β-OHB level (Fig. 1b), indicating that the regulation of β-OHB production in AD brain is impaired. H3k9bhb, a novel histone modification mark induced by ketogenesis-generated β-OHB, which mediates chromatin opening^29^. Mouse experiments showed that lysine β-hydroxybutyrylation (Kbhb) and H3k9bhb levels were significantly reduced in the hippocampus of 9-month-old 3xTg-AD mice compared with age-matched WT mice (Fig. 1c, d), which were significantly co-localized with neuronal marker NeuN (Fig. 1e, f). Compared with healthy individuals, the level of H3K9bhb in brain tissue of patients with AD was significantly decreased (Fig. 1g–i). The above results support that β-OHB and H3K9bhb are both reduced in the AD background, but whether they are involved in the regulation of AD synaptic function remains unclear.

### β-OHB supplementation upregulates the expression of H3K9bhb in the hippocampus of 3xTg-AD mice

**Fig. 2:**
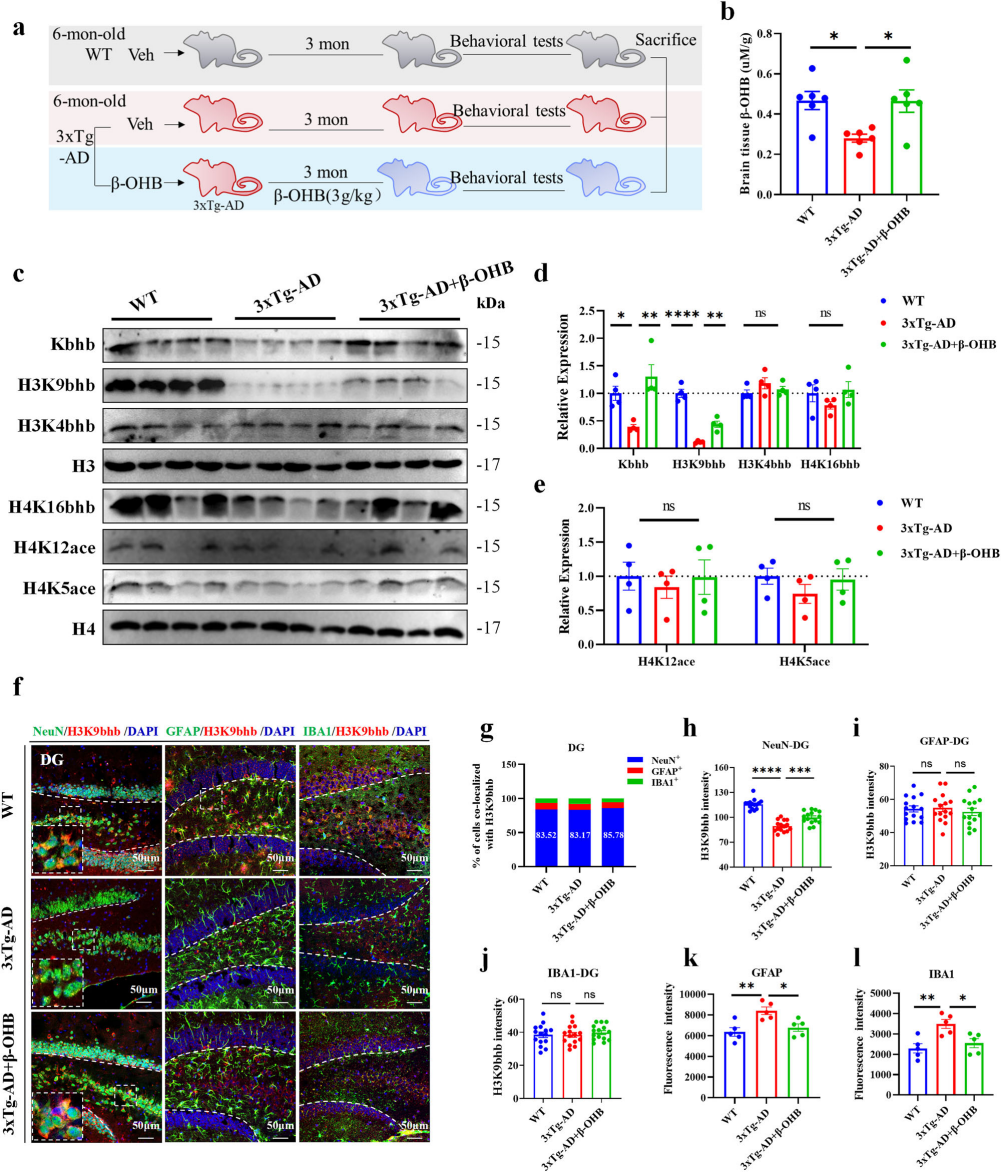
β-OHB supplementation increases H3K9bhb level in the hippocampus of 3xTg-AD mice. **a**, The experimental design for β-OHB-treated 3xTg-AD mice. **b**, After supplementing with β-OHB, the levels of β-OHB in the hippocampus of 9-month-old 3xTg-AD mice were detected, *n* = 6 per group. **c**,**d**, The western blotting experiments were used to detect (**c**) and quantitatively analyze (**d**) the levels of pan- and site-specific hippocampal β-hydroxybutylation (H3K9, H3K4, H4K16), *n* = 4 per group. **c**,**e**, The levels of acetylation sites such as histones H4K12 and H4K5 were detected (**c**) and analyzed (**e**) by Western blotting experiments, *n* = 4 per group. **f**–**l**, The immunofluorescence assay measures the levels of H3K9bhb in neuronal cells, astrocytes and microglia (**f**), along with % of cells co-localized with H3K9bhb (**g**), H3K9bhb fluorescence in hippocampal DG region neurons (**h**), microglia (**i**) and glial cells (**j**) across three experimental groups, *n* = 15 cells (**h**–**j**), fluorescence intensity quantification of GFAP (**k**), fluorescence intensity quantification of IBA1 (**l**), *n* = 5 per group. Scale bar, 50 μm. Data are shown as mean ± s.e.m. One-way ANOVA followed by Bonferroni’s post hoc test for **b**, **d**, **e** and **h**–**l**. ^*^P < 0.05, ^**^P < 0.01, ^***^P < 0.001, ^****^P < 0.0001; ns, not significant.

To confirm that H3K9bhb is directly regulated by β-OHB in 3xTg-AD mice, we supplemented 3xTg-AD mice with β-OHB from 6 months to 9 months and found that β-OHB supplementation significantly increased the level of β-OHB in the brain of 3xTg-AD mice (Fig. 2a, b). Further molecular experiments showed that the protein levels of Kbhb and H3K9bhb was significantly increased without significant effect on histone acetylation with β-OHB supplementation (Fig. 2c–e), which was consistent with the previous results of fasting^30^. To assess cell type-specific changes in histone β-hydroxybutyrylation, we performed immunofluorescence co-staining of H3K9bhb with markers for neurons (NeuN), astrocytes (GFAP) and microglia (Iba1). Quantitative analysis revealed that H3K9bhb was predominantly localized in neurons, with 83.14–89.40% of H3K9bhb-positive cells co-localizing with NeuN (Fig. 2f, g and Supplementary Fig. 1a,b,f,g,k,l). Furthermore, H3K9bhb immunoreactivity was significantly reduced in the hippocampal neurons of 3xTg-AD mice compared with WT mice, and this deficit was rescued by β-OHB supplementation, which significantly enhanced H3K9bhb expression in neurons of 3xTg-AD mice (Fig. 2h and Supplementary Fig. 1c,h,m). Notably, no significant changes in H3K9bhb immunoreactivity were observed in hippocampal glial cells of 3xTg-AD mice compared with WT controls, and β-OHB supplementation did not elicit a notable alteration in H3K9bhb levels in glial cells (Fig. 2I, j and Supplementary Fig. 1d,e,i,j,n,o). Collectively, these data indicate that H3K9bhb predominantly functions in neurons and may play a key role in the epigenetic regulation of gene expression in AD. Interestingly, we observed significant microglial and astrocytic activation in the hippocampus of 3xTg-AD mice; this neuroinflammatory response was robustly suppressed following β-OHB supplementation (Fig. 2k, l), aligning with earlier findings^21^.

### The β-OHB supplementation restores the level of axon guidance related genes by increasing H3K9bhb in middle‑aged 3xTg-AD mice

To explore the effect of β-OHB/H3K9bhb on the transcriptome of 3xTg-AD mice, we first examined hippocampal bulk RNA sequencing in WT, 3xTg-AD and 3xTg-AD+β-OHB mice. The differentially expressed genes were defined as *P* value < 0.05 and |fold change| >1.1. The data analysis showed that a total of 1,276 genes were significantly differentially expressed in 3xTg-AD versus WT mice, of which, 762 were downregulated and 514 were upregulated (Supplementary Fig. 2a). Furthermore, KEGG analysis showed that downregulated genes were mainly enriched in protein digestion and absorption, electron transport chain, TGF-β signaling pathway, lysosome and axon guidance signaling pathways, and the upregulated genes were mainly enriched in gap junction. neuroactive ligand–receptor interaction, PI3K–Akt–mTOR signaling pathway, morphine addiction, autophagy signaling pathways (Supplementary Fig. 2b, c). However, data analysis showed that a total of 422 genes were significantly differentially expressed in 3xTg-AD+β-OHB versus 3xTg-AD mice, of which, 231 were downregulated and 191 were upregulated (Supplementary Fig. 2d). Furthermore, KEGG showed that the downregulated genes were mainly enriched in neuroactive ligand–receptor interaction, calcium signaling pathway, cGMP–PKG signaling pathway, adipogenesis genes and PI3K–Akt–mTOR signaling pathways, and the upregulated genes were mainly enriched in axon guidance, adipogenesis genes, dopaminergic neurogenesis, regulation of actin cytoskeleton and cytokine–cytokine receptor interaction signaling pathway (Supplementary Fig. 2e, f).

**Fig. 3:**
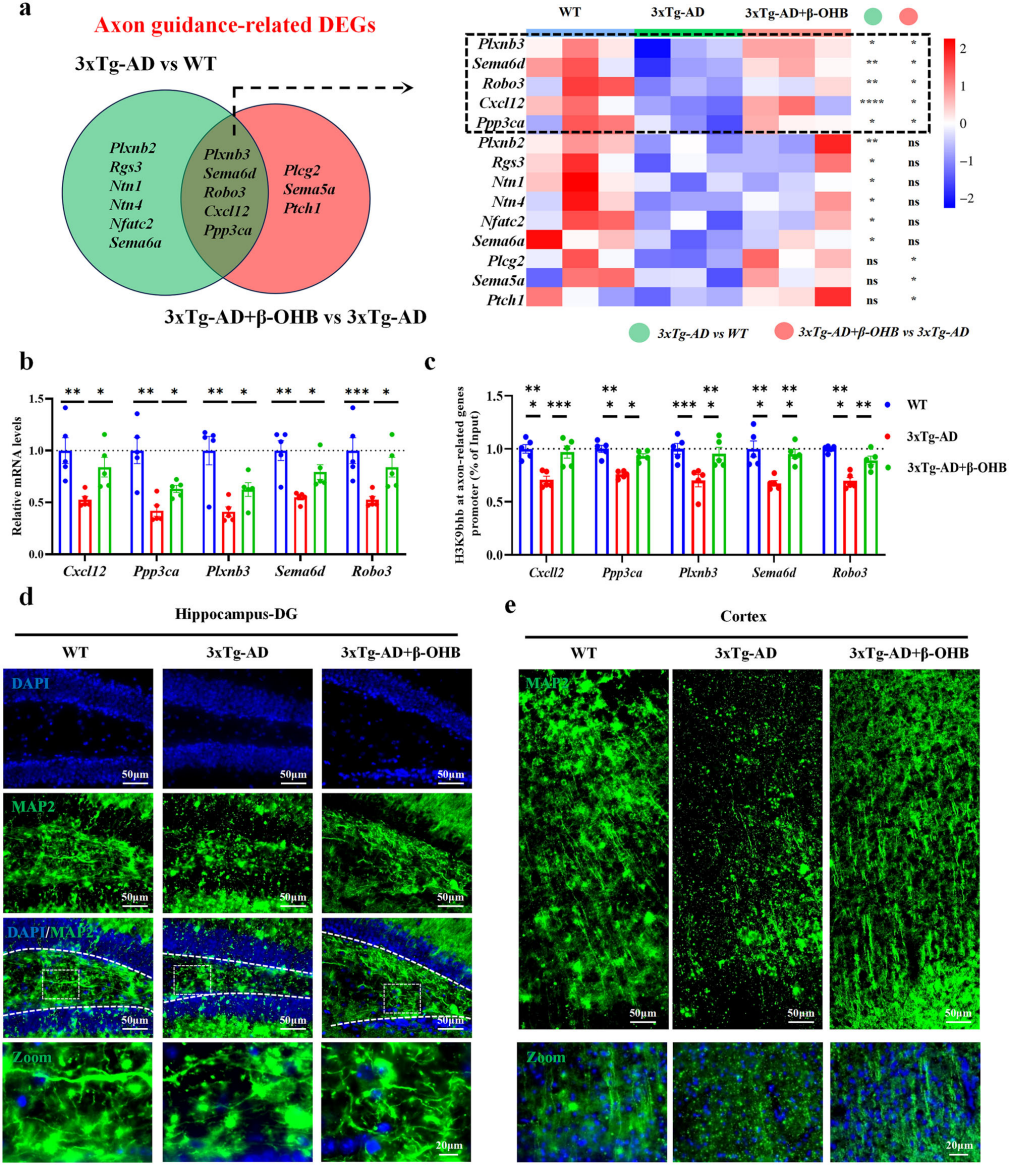
H3K9bhb was significantly reduced in the promoter region of axon-guidance related genes in 3xTg-AD mice, which could be improved by supplementation of β-OHB. **a**, Compared with WT mice, axon-guidance related genes were significantly downregulated in 9-month-old 3xTg-AD mice, whereas axon-guidance related genes were significantly upregulated after β-OHB supplementation, *n* = 3 per group. **b**, The RT–qPCR validation of axonal guidance related genes after supplementation with β-OHB, *n* = 5 per group. **c**, ChIP–qPCR was used to detect the enrichment of H3K9bhb in the promoter region of axonal guidance related genes in mice neurons, *n* = 5 per group. **d**,**e**, β-OHB supplementation can significantly improve 3xTg-AD hippocampal (**d**) and cortical axonal (**e**) deficits. Scale bar, 50 μm. Data are shown as mean ± s.e.m. One-way ANOVA followed by Bonferroni’s post hoc test for **b** and **c**.^*^P < 0.05, ^**^P < 0.01, ^***^P < 0.001, ^****^P < 0.0001; ns, not significant.

Interestingly, the synaptic plasticity related signaling pathway, namely, axonal guidance, was significantly inhibited in 3xTg-AD mice, which was significantly reversed after β-OHB supplementation (Supplementary Fig. 2b, f). A Venn analysis showed that five axon guidance related genes were significantly decreased in 3xTg-AD mice, which was significantly increased after β-OHB supplementation (Fig. 3a). These results indicate that β-OHB supplementation improves synaptic plasticity via promoting axon guidance related genes in the middle-aged 3xTg-AD mice.

β-OHB promotes chromatin opening and gene expression by increasing H3K9bhb levels^29^. To test whether β-OHB supplementation restores the levels of axon guidance-related genes by increasing H3K9bhb, we performed the following experiments. Consistent with the transcriptional results, reverse-transcription quantitative PCR (RT–qPCR) showed that the expression levels of *Cxcl12*, *Ppp3ca*, *Plxnb3*, *Sema6d* and *Robo3* were significantly decreased in the 3xTg-AD versus WT, which were significantly reversed after administration of β-OHB (Fig. 3b). Next, after neuron-specific sorting, we perform ChIP–qPCR analysis showing that 3xTg-AD mice exhibited a low enrichment of H3k9bhb at the promoters of axon guidance-related genes, whereas β-OHB replenishment enhanced H3K9bhb and its enrichment in the promoter regions of axon guidance-related genes in mice neurons (Fig. 3c). Correspondingly, the level of axon biomarker MAP2 was significantly increased after β-OHB supplementation, and the axon morphology was significantly improved in 3xTg-AD after β-OHB supplementation (Fig. 3d, e). Collectively, these results suggest that β-OHB supplementation upregulates axon guidance-related gene expression by increasing H3K9bhb levels and its enrichment at their promoters.

### The β-OHB supplementation restores the level of synapse-related genes by increasing H3K9bhb in middle‑aged 3xTg-AD mice

**Fig. 4:**
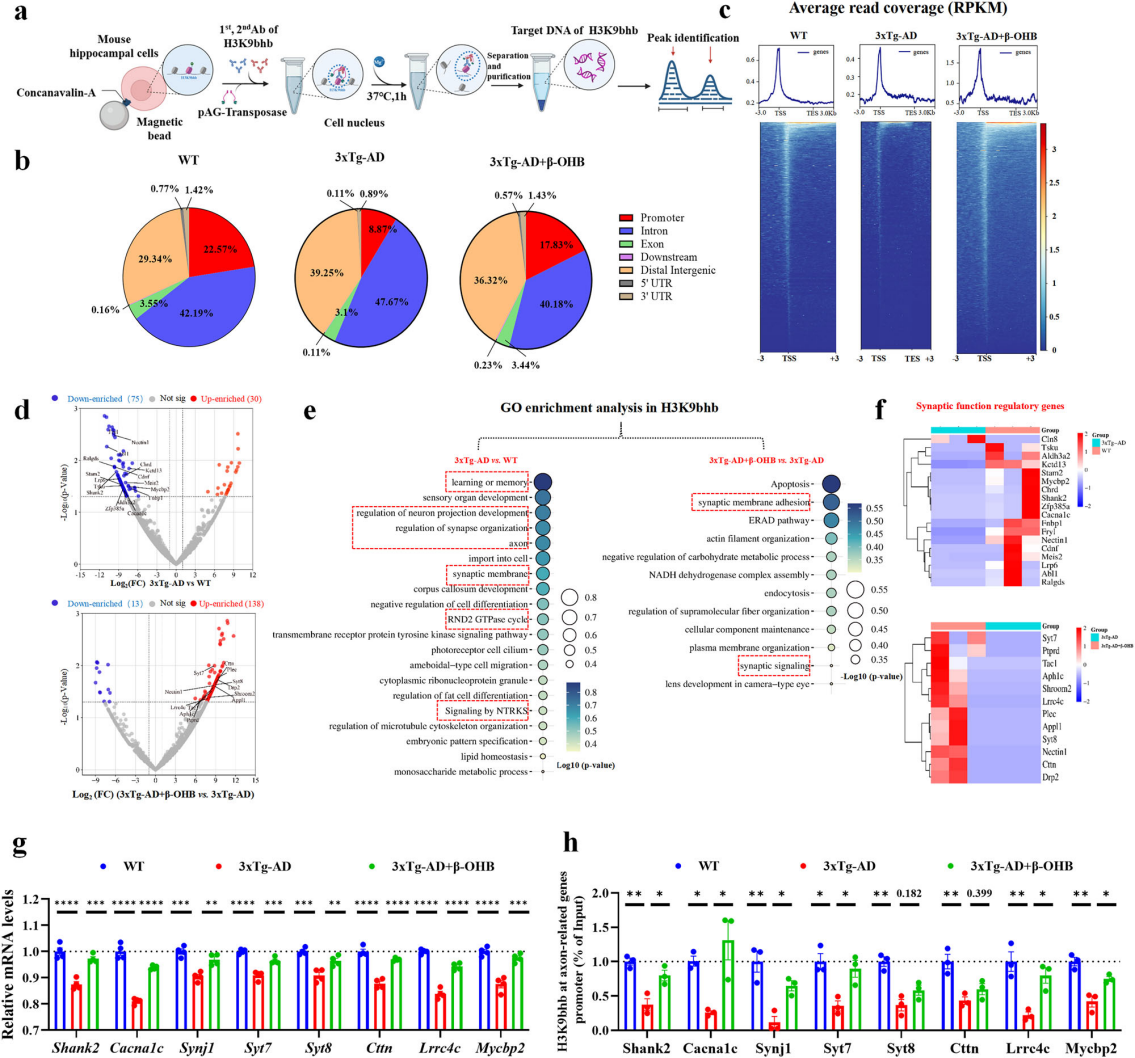
β-OHB replenishment enhanced H3K9bhb enrichment in the promoter regions of axon and synapse-related genes. **a**, The CUT&Tag sequencing schematic for analyzing transcriptome changes regulated by H3K9bhb, *n* = 3 per group. **b**, The genome-wide distribution of H3K9bhb-binding peaks in the hippocampus of from WT, 3xTg-AD and 3xTg-AD+β-OHB mice. **c**, The binding density of H3K9bhb was visualized by deepTools: the heat map presents the CUT&Tag tag counts on the different H3K9bhb binding peaks in hippocampus extracts between WT, 3xTg-AD and 3xTg-AD+β-OHB mice, ordered by signal strength. **d**, Differential enrichment of H3K9bhb at the promoter region of target genes. **e**, A biological processes analysis of H3K9bhb binding peaks at candidate differentially expressed target genes. **f**, Differential enrichment of H3K9bhb at the promoter region of axon and synapse-related genes. **g**, The mRNA levels of the synapse-related genes, *n* = 4 per group. **h**, The enrichment of H3K9bhb on the promoters of the axon-related genes, *n* = 3 per group. Data are shown as mean ± s.e.m. One-way ANOVA followed by Bonferroni’s post hoc test for **g** and **h**.^*^P < 0.05, ^**^P < 0.01, ^***^P < 0.001, ^****^P < 0.0001; ns, not significant.

Epigenetic modifications of histones can affect the transcriptional activation or inhibition of target genes. To explore the transcriptional regulatory role of H3K9bhb in AD, we used H3K9bhb antibody and CUT&Tag kits to identify candidate genes for H3K9bhb regulation (Fig. 4a). The results showed that compared with WT, the enrichment of H3K9bhb in the promoter region of the target gene in the 3xTg-AD mice hippocampus was significantly reduced (3xTg-AD: 8.87%; WT: 22.57%), whereas after supplementing with β-OHB, the enrichment of H3K9bhb in the promoter region of the target gene was significantly increased (3xTg-AD+β-OHB: 17.83%; 3xTg-AD: 8.87%). In addition, the peak of H3K9bhb in the target gene was also significantly increased in the 3xTg-AD+β-OHB group (Fig. 4b, c). Moreover, the repeated H3K9bhb CUT&Tag results also showed that compared with WT, the enrichment of H3K9bhb in the promoter region of the target gene in the 3xTg-AD mice hippocampus was significantly reduced (3xTg-AD: 3.80%; WT: 17.50%), whereas after supplementing with β-OHB, the enrichment of H3K9bhb in the promoter region of the target gene was significantly increased (3xTg-AD+β-OHB: 10.08%; 3xTg-AD: 3.80%), and the peak of H3K9bhb in the target gene was also significantly increased in the 3xTg-AD+β-OHB group (Supplementary Fig. 3).

Correspondingly, our analysis of H3K9bhb binding at promoter regions identified 105 differentially enriched target genes in 3xTg-AD mice compared with WT. Of these, 75 genes exhibited down-enrichment and 30 showed up-enrichment (Fig. 4d); however, the comparison between the 3xTg-AD+β-OHB and 3xTg-AD groups revealed 151 differentially bound target genes, with a striking reversal in the pattern: 138 genes were up-enriched and only 13 were down-enriched after β-OHB treatment (Fig. 4d). The biological process analysis showed that the different H3K9bhb-enriched genes in 3xTg AD versus WT mice were mainly enriched in learning and memory, regulation of synapse organization and axon and synaptic membrane; the different H3K9bhb-enriched genes in 3xTg-AD+β-OHB versus 3xTg AD were significantly enriched in synaptic membrane adhesion and synaptic signaling (Fig. 4e). Axon- or synapse-related genes, including voltage-dependent L-type calcium channel subunit alpha-1C (Cacna1c), SH3 and multiple ankyrin repeat domains protein 2 (Shank2), synaptojanin-1 (Synj1), synaptotagmin-7 (Syt7), synaptotagmin-8 (Syt8), cortactin (Cttn), leucine-rich repeat-containing protein 4C (Lrrc4c) and Myc-binding protein 2 (Mycbp2), showed that the levels of H3K9bhb at the promoters of these genes were reduced in 3xTg-AD versus WT but increased in 3xTg-AD+β-OHB versus 3xTg-AD (Fig. 4f). Consistent with CUT&Tag data, RT–qPCR and ChIP–qPCR analyses demonstrated that β-OHB supplementation significantly enhanced both the transcription of key axonal and synaptic genes and the enrichment of H3K9bhb at their promoters (Fig. 4g, h). Collectively, these results suggest that β-OHB supplementation enhances the transcription of several known axon- and synapse-related genes in AD through H3K9bhb modification.

**Fig. 5:**
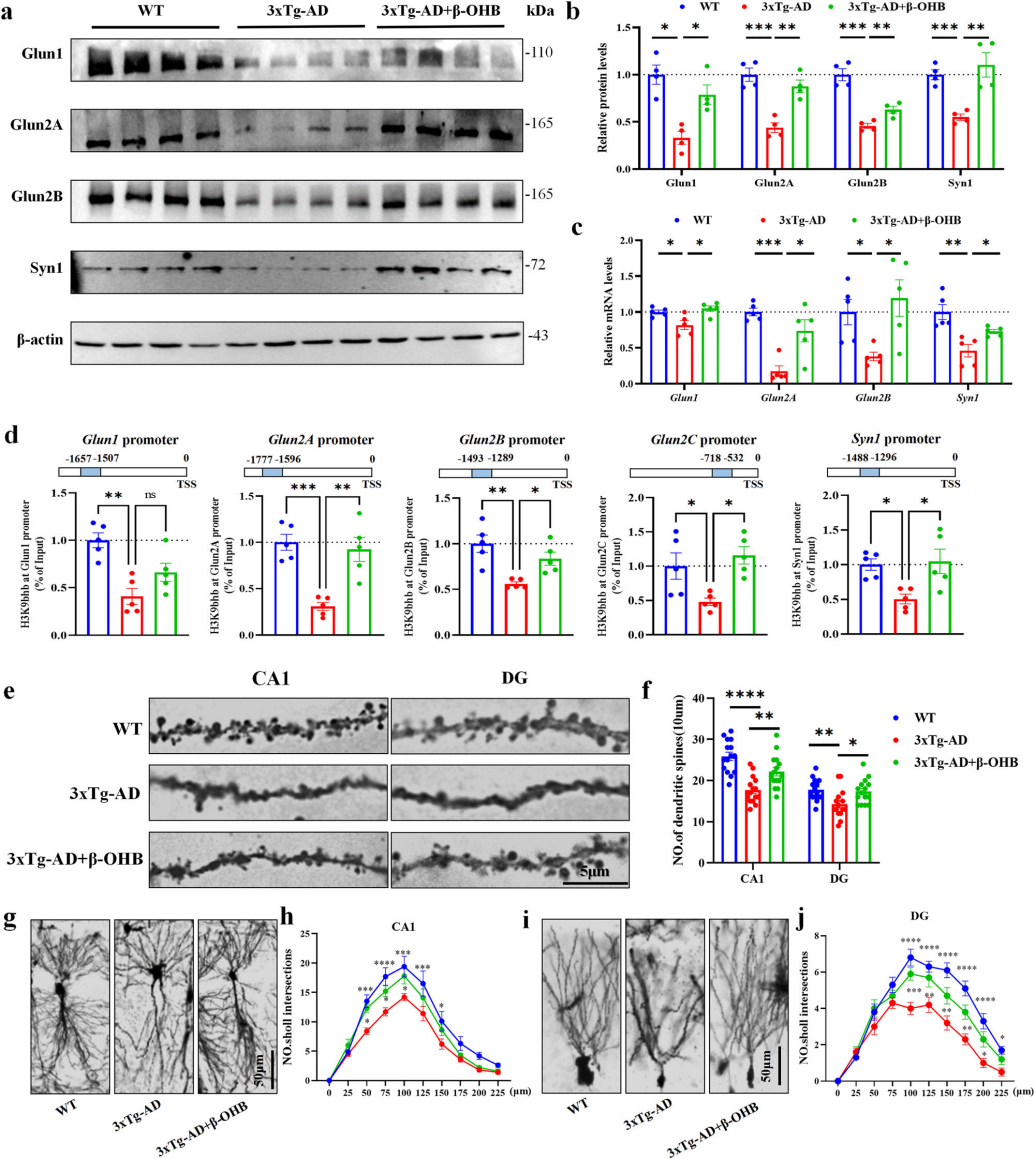
The β-OHB supplementation-restored synaptic plasticity via the H3K9bhb-mediated expression. **a**,**b**, The representative immunoblots (**a**) and quantitative analyses (**b**) of Glun1, Glun2A, Glun2B and Syn1 in the hippocampus of the WT, 3xTg-AD and 3xTg-AD+β-OHB mice, *n* = 4 per group. **c**, RT–qPCR assays mRNA expression of the *Glun1, Glun2A, Glun2B* and *Syn1* in the hippocampus of the WT, 3xTg-AD and 3xTg-AD+β-OHB mice, *n* = 5 per group. **d**, ChIP–qPCR analysis of the enrichment of H3K9bhb at *Glun1, Glun2A, Glun2B, Glun2C* and *Syn1* promoters in the hippocampus of the WT, 3xTg-AD and 3xTg-AD+β-OHB mice, *n* = 5 per group. **e**,**f**, Supplementing with β-OHB could increase the density of 3xTg-AD dendritic spines detected by Golgi-cox staining; the representative images (**e**) and quantitative analysis (**f**) of spine, *n* = 5 per group, three fields per mice. Scale bar, 5 μm. **g**–**j**, The Sholl analysis showed the synaptic complexity of neurons after supplementing with β-OHB in 3xTg-AD mice; the representative images (**g** and **i**) and the quantitative analysis (**h** and **j**), *n* = 5 per group, two fields per mice. Scale bar, 50 μm. Data are shown as mean ± s.e.m. One-way ANOVA followed by Bonferroni’s post hoc test for **b**–**d** and **f**. Two-way ANOVA followed by Bonferroni’s post hoc test for **i** and **k**.^*^P < 0.05, ^**^P < 0.01, ^***^P < 0.001, ^****^P < 0.0001; ns, not significant.

In addition, NMDA receptor subunits (NMDARs) regulate the production and maintenance of long-term potentiation and long-term depression, which are crucial for synaptic plasticity and memory^31^. The western blot analysis showed that the expression of NMDARs (Glun1, Glun2A, Glun2B) and synaptic vesicle-related protein Syn1 were significantly decreased in 3xTg-AD mice, whereas they were significantly increased after β-OHB supplementation (Fig. 5a, b). To test whether β-OHB supplementation restores the level of NMDARs and *Syn1* by increasing H3K9bhb, we performed the following experiments. First, RT–qPCR showed that the mRNA expression levels of *Glun1*, *Glun2A*, *Glun2B* and *Syn1* were significantly decreased in the 3xTg-AD versus WT, which were reversed after administration of β-OHB (Fig. 5c). Next, ChIP–qPCR analysis showed that 3xTg-AD mice exhibited a low enrichment of H3k9bhb on the promoters of NMDARs-related genes and *Syn1*, whereas β-OHB replenishment enhanced H3K9bhb levels and its enrichment in the promoter regions of NMDARs-related genes and *Syn1* (Fig. 5d). Finally, we analyzed the number of neuronal branches and dendritic spines in the experimental animals by observing synaptic morphological changes through Golgi-Cox staining. Compared with the 3xTg-AD group, the 3xTg-AD+β-OHB group had more dendritic spines (Fig. 5e, f) and longer neuronal branching lengths (Fig. 5g–j and Supplementary Fig. 4) in the CA1 and DG regions. These results suggest that β-OHB replenishment enhanced H3K9bhb and its enrichment in the promoter regions of NMDARs-related genes and synaptic vesicle-related gene *Syn1*, thereby improving synaptic activity.

### The β-OHB supplementation improves learning and memory function in middle-aged 3xTg-AD

**Fig. 6:**
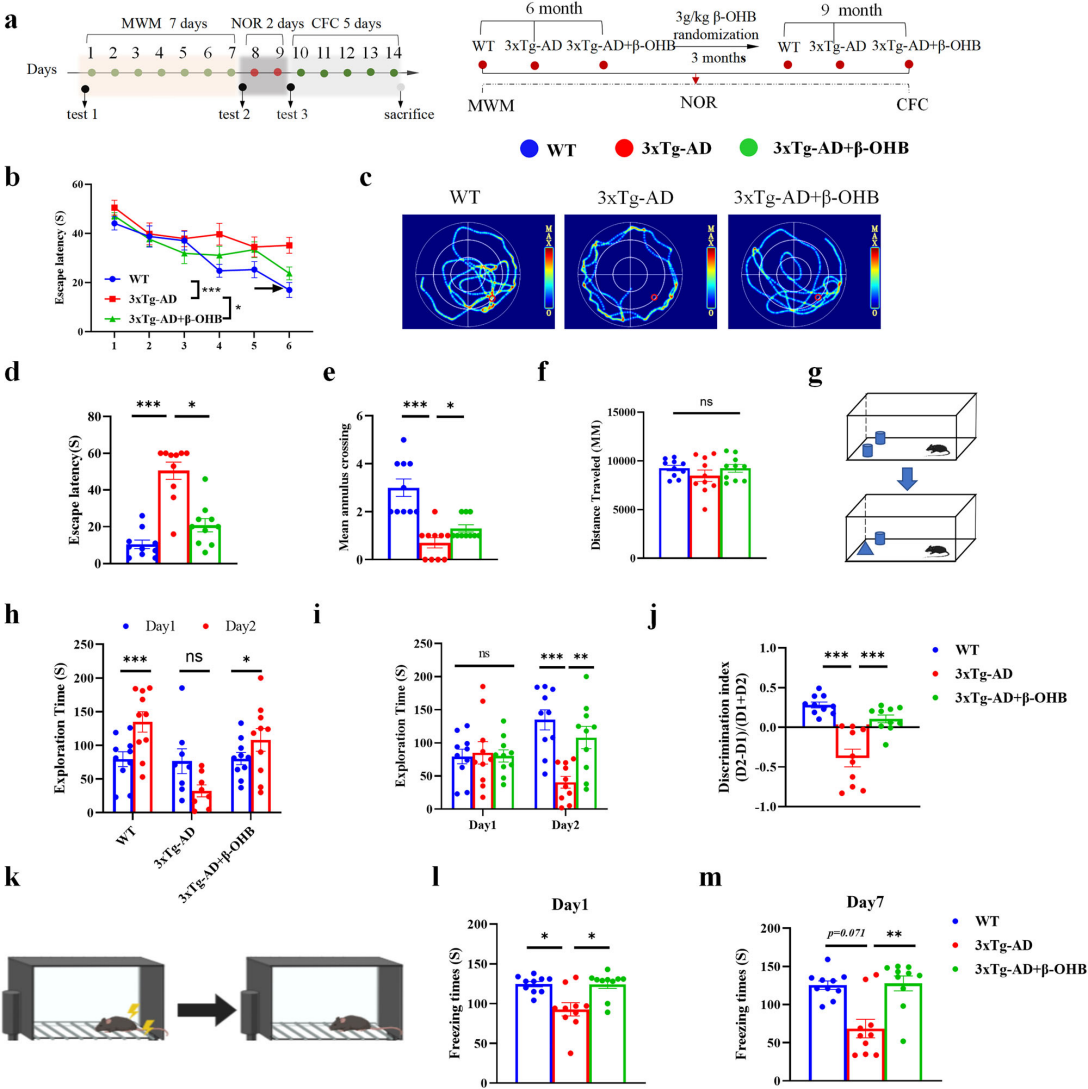
The β-OHB supplementation improves learning and memory function in middle-aged 3xTg-AD. **a**, The experimental workflow of the in vivo study, *n* = 10 per group. **b**–**f**, The MWM was used to detect the effect of β-OHB treatment on the spatial learning and memory function of 3xTg-AD mice fed with 3 g/kg β-OHB; the escape latency to the hidden platform in the training phase (**b**), the swimming pathway traveled to locate the platform on day 7 (**c**), the escape latency of day 7 (**d**), the counts of the original position of the platform crossing (**e**) and the average swimming distance of the mice (**f**). **g**–**j**, The NOR test was also used to access the learning and memory ability of mice treated with 3 g/kg β-OHB; the change of exploration time of three groups between day 1 and day 2 (**h**), exploration time to novel object between day 1 and 2 (**i**) and discrimination index of new object (**j**). **k**–**m**, The effect of foot shock-induced contextual fear freezing test (**k**). Freeze time on the first (**l**) and seventh days (**m**). Data are shown as mean ± s.e.m. Unpaired *t*-test for **h**. One-way ANOVA followed by Bonferroni’s post hoc test for **d**–**f**, **i**, **j**, **l** and **m**. Two-way ANOVA followed by Bonferroni’s post hoc test for **b**.^*^P < 0.05, ^**^P < 0.01, ^***^P < 0.001; ns, not significant.

To assess the ability of learning and memory in middle-aged 3xTg-AD mice, we performed the Morris water maze (MWM), novel object recognition (NOR) and fear condition tests (Fig. 6a). During the training trials of MWM experiment (1–6 days), 3xTg-AD mice spent more time to find the target platform, whereas β-OHB supplementation significantly shortened the time to find the target platform, which indicated that β-OHB significantly improves the learning ability of 3xTg-AD mice (Fig. 6b). However, in the probe trial, the 3xTg-AD group spent more time to first find the target platform and made fewer entries to the target quadrant, whereas those with β-OHB supplementation shortened the time to first find the target platform and made more entries to the target quadrant; these results indicate that β-OHB significantly improves the memory ability of 3xTg-AD mice (Fig. 6c–e). Furthermore, no significant differences in swimming total distance traveled were observed among the groups, thereby ruling out the potential confounding effects of motor performance on the assessment of learning and memory (Fig. 6f). In the NOR test, 3xTg-AD spent less time exploring new objects, whereas supplementation with β-OHB significantly increased the time on exploring new objects of 3xTg-AD mice (Fig. 6g–i). Correspondingly, the 3xTg-AD mice showed a significant reduction in the discrimination index of novel objects compared with the WT group, which was significantly increased after β-OHB supplementation (Fig. 6j). In the fear condition test, the freezing time in 3xTg-AD was significantly reduced both day 1 and day 7 after electrical stimulation, whereas β-OHB supplementation improved the number of freezing, indicating that β-OHB ameliorates not only short-term memory but also long-term memory in 3xTg-AD mice (Fig. 6k–m). Together, β-OHB could significantly improve cognitive function in middle-aged 3xTg-AD mice.

### HMGCS2 expression decreases in the brains of middle-aged 3xTg-AD mice and patients with AD as well as Aβ-treated N2a cells and 3xTg-AD primary neurons

**Fig. 7:**
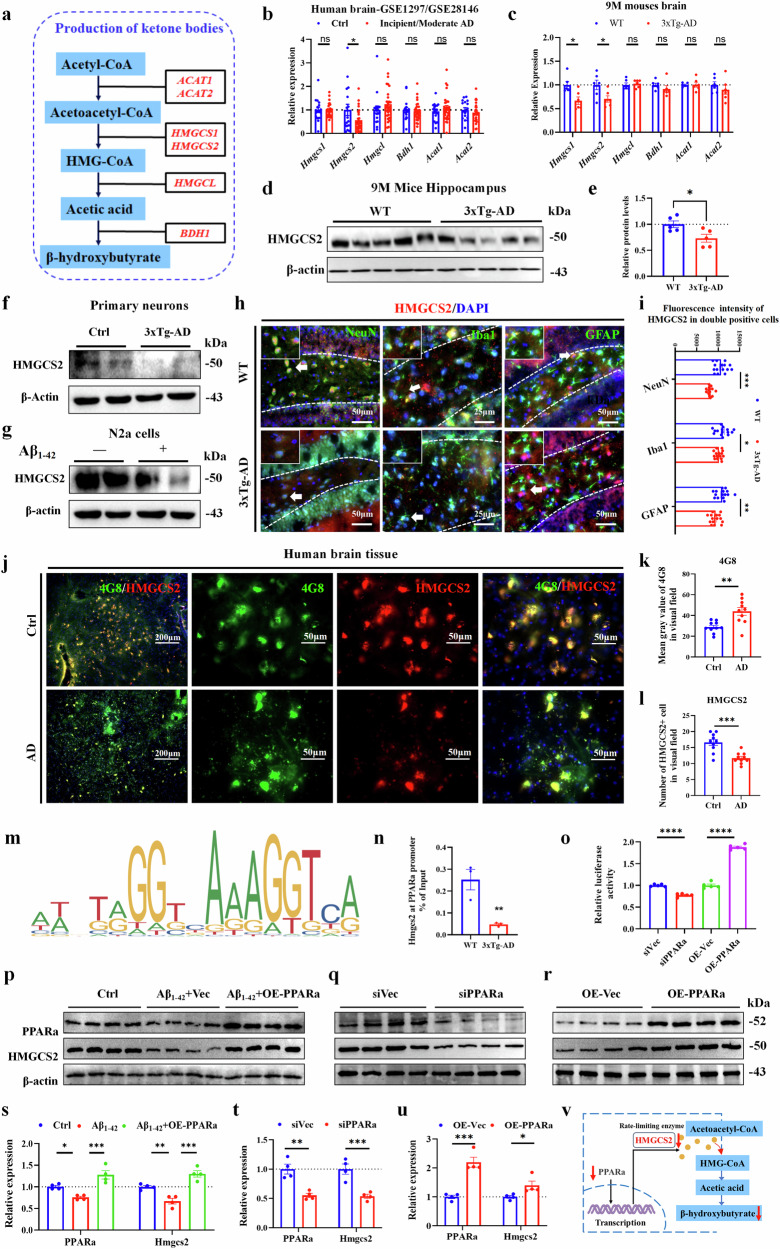
HMGCS2 expression decreases in the brains of middle-aged 3xTg-AD mice and patients with AD, as well as Aβ-treated N2a cells and 3xTg-AD primary neurons. **a**, The β-OHB formation process and related enzymes. **b**,**c**, The expression of β-OHB-producing enzymes in the brains of patients with AD (GSE1297/GSE28146, Ctrl *n* = 17, incipient/moderate AD *n* = 30) (**b**) and 3xTg-AD mice (**c**) *n* = 6 per group. **d**–**g**, The representative immunoblots (**d**) and quantitative analyses of HMGCS2 in hippocampus (**e**), *n* = 5 per group, and representative immunoblots (**f**) and quantitative analyses of primary neurons of 3xTg-AD mice (**g**) and Aβ_1-42_-treated N2a cells. **h**,**i**, HMGCS2 was co-localized with neurons (NeuN), astrocytes (GFAP) and microglia (IBA1) and measured using immunofluorescence staining, *n* = 5 per group, three fields per mice, scale bar, 50 μm (**h**), and the quantitative fluorescence analysis (**i**). **j**,**l**, Immunofluorescence (**j**) was used to detect the expression of Aβ-4G8 in the brain of a patient with AD (**k**), and the expression of HMGCS2 in the brain of patients with AD was detected by immunofluorescence (**l**), *n* = 4 per group, ten fields per group. Scale bar, 50 μm. **m**, The putative PPARα binding motif on the HMGCS2 promoter, predicted by JASPAR. **n**, The enrichment of PPARα on the HMGCS2 promoter, *n* = 3 per group. **o**, The dual-luciferase reporter assay results, *n* = 5 per group. **p**–**u**, western blot analysis validating HMGCS2 expression levels: after PPARα overexpression in N2a-Aβ_1-42_ cells, *n* = 4 per group (**p** and **s**), and after PPARα knockdown (**q** and **t**) or overexpression (**r** and **u**) in N2a cells, *n* = 4 per group (**q**, **r**, **t** and **u**). **v**, A schematic diagram of the proposed mechanism. Data are shown as mean ± s.e.m. Unpaired *t*-test for **b**, **c**, **e**, **i**, **k**, **l**, **t** and **u**. One-way ANOVA followed by Bonferroni’s post hoc test for **n**, **o** and **s**. ^*^P < 0.05, ^**^P < 0.01, ^***^P < 0.001; ns, not significant.

To explore the specific mechanism underlying the reduction of β-OHB production in AD, we performed GEO data (GSE1297/GSE28146) analysis and RT–qPCR experiments and found that the transcription level of HMGCS2, a key enzyme for β-OHB production, was significantly reduced in patients with AD and 3xTg-AD mice (Fig. 7a–c). Further, western blotting experiments showed that HMGCS2 protein expression was significantly reduced in the hippocampus and primary neurons of 3xTg-AD mice and Aβ_1-42_-treated N2a cells (Fig. 7d–g and Supplementary Fig. 5a, b). The immunohistochemistry of HMGCS2 and colocalization of HMGCS2 signals with NeuN, GFAP and IBA1 were observed across all subregions of the hippocampus, suggesting that HMGCS2 exhibited extensive expression across all neural cells and was significantly reduced in 3xTg-AD mice (Fig. 7h–i and Supplementary Fig. 5c, d). Immunofluorescence assay showed that HMGCS2 was significantly decreased in AD brain tissues (Fig. 7j–l). These results suggested that HMGCS2 expression was significantly reduced in the brain tissues of patients with AD and 3xTg-AD mice, as well as Aβ-treated N2a cells and 3xTg-AD primary neurons. The low expression of HMGCS2 may inhibit the production of β-OHB, thereby leading to cognitive impairment by reducing the enrichment of H3K9bhb in the promoter region of synapse-related genes.

To elucidate the mechanism underlying HMGCS2 downregulation in AD, we first used the JASPAR database to predict potential transcription factors regulating HMGCS2 (Supplementary Fig. 6a). Subsequent real-time PCR validation identified PPARα as the only transcription factor significantly downregulated in the hippocampus of 3xTg-AD mice (Supplementary Fig. 6b). To further validate PPARα‘s direct regulatory role, we performed ChIP–qPCR and found significantly reduced PPARα enrichment at the HMGCS2 promoter in 3xTg-AD mice (Fig. 7m, n). Dual-luciferase reporter assays confirmed that either downregulation or upregulation of PPARα correspondingly suppressed or activated HMGCS2 transcription (Fig. 7o). Consistently, in Aβ_1-42_-treated N2a cells, PPARα overexpression rescued HMGCS2 expression (Fig. 7p, s). Furthermore, PPARα downregulation significantly suppressed HMGCS2, whereas its overexpression enhanced HMGCS2 expression in N2a cells (Fig. 7q, r, t, u). Collectively, these results demonstrate that HMGCS2 expression is directly regulated by PPARα, whose downregulation in AD consequently impairs β-OHB production in the brain (Fig. 7v).

### The HMGCS2 upregulation enhances the synaptic plasticity-associated genes by increasing the expression of H3K9bhb in 3xTg-AD mice

**Fig. 8:**
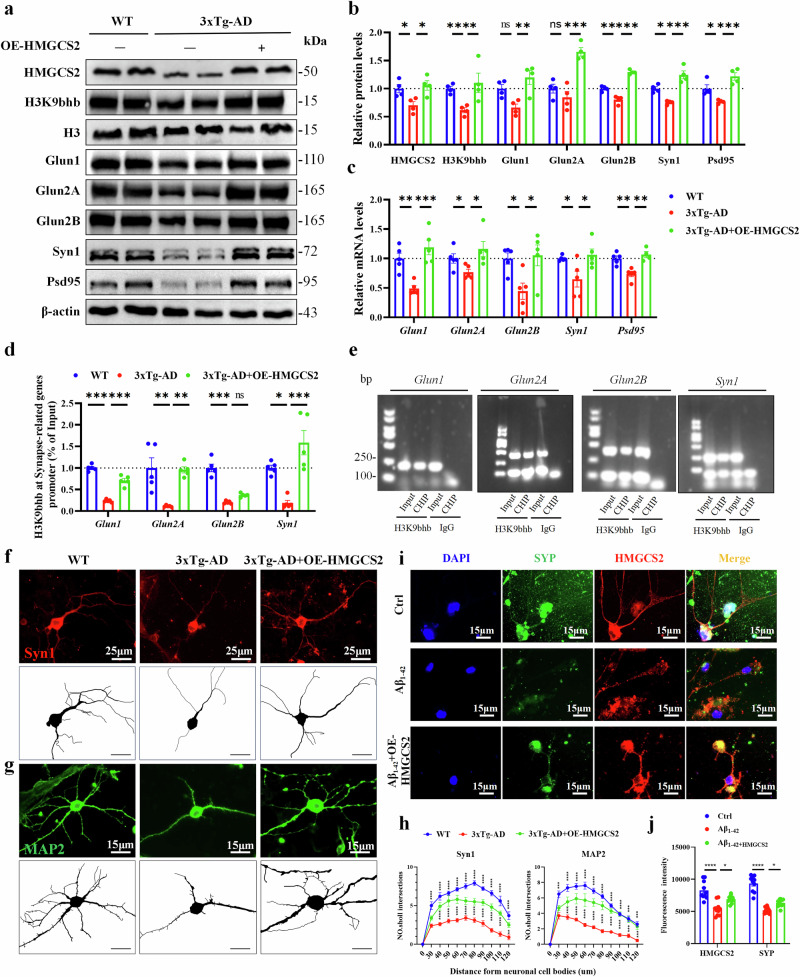
The HMGCS2 upregulation enhances the synaptic plasticity-associated genes by increasing the expression of H3K9bhb in primary neurons of 3xTg-AD mice. **a**–**c**, The HMGCS2 upregulation promotes the protein (**a** and **b**) and mRNA (**c**) expression of H3K9bhb, Glun1, Glun2A, Glun2B, Syn1 and PSD95, *n* = 4 or 5 per group. **d**,**e**, ChIP–qPCR analyses of the enrichment of H3K9bhb at *Glun1, Glun2A, Glun2B* and *Syn1* promoters in the primary neurons of the WT, 3xTg-AD and 3xTg-AD + HMGCS2 mice *n* = 5 per group (**d**) and representative gel images from ChIP–qPCR assays (**e**). **f**–**j**, The HMGCS2 upregulation promotes the expression of Syn1 (scale bar, 25 μm) (**f**); *n* = 10 cells per group in MAP2 immunofluorescence (**g**) and quantitative analysis (**h**), *n* = 10 cells per group and SYP (**i** and **j**), *n* = 10 cells per group, scale bar, 15 μm. Data are shown as mean ± s.e.m. One-way ANOVA followed by Bonferroni’s post hoc test for **b**–**d** and **j**. Two-way ANOVA followed by Bonferroni’s post hoc test for **h**.^*^P < 0.05, ^**^P < 0.01^, ***^P < 0.001, ^****^P < 0.0001; ns, not significant.

HMGCS2 is a key rate-limiting enzyme for β-OHB production, which may be closely related to H3K9bhb-mediated synaptic related gene expression. To demonstrate whether HMGCS2 upregulation can restore synaptic plasticity of 3xTg-AD mice by enhancing H3K9bhb, we conducted a series of experiments. First, we conducted western blotting experiments to detect the protein expression levels of HMGCS2 and H3K9bhb after overexpressing HMGCS2. The results showed that the H3K9bhb level of the HMGCS2 upregulated group and the WT group was higher than in the 3xTg-AD primary neuron group (Fig. 8a, b). Moreover, synapse-associated proteins were significantly decreased in 3xTg-AD, which could be significantly reversed by HMGCS2 upregulation (Fig. 8a–c). Then, we designed primers targeting the promoter regions of genes (*Glun1*, *Glun2A*, *Glun2B*, *Syn1*) and measured the occupancy of H3K9bhb at these regions through ChIP–qPCR assays. The analysis showed that the enrichment of H3K9bhb at *Glun1*, *Glun2A*, *Glun2B* and *Syn1* was significantly reversed by HMGCS2 upregulation (Fig. 8d, e). In addition, it was found that HMGCS2 upregulation could significantly improve the synaptic complexity (Syn1 and synaptophysin (SYP)) and axonal function (MAP2) of 3xTg-AD primary neurons (Fig. 8f–j), indicating that the synaptic plasticity of AD primary neurons was significantly enhanced. To clarify the specific role of HMGCS2 in regulating β-OHB production and H3K9bhb levels while excluding potential off-target effects, we performed additional experiments using an enzymatically inactive form of HMGCS2. We found that the overexpression of the WT HMGCS2 significantly increased the levels of both β-OHB and H3K9bhb, whereas the enzymatically dead mutant had no such effect (Supplementary Fig. 7a–c). Consistent with these biochemical changes, WT HMGCS2 overexpression significantly upregulated the expression of synaptic genes (*Glun1*, *Glun2a*, *Glun2b*, *Syn1*, *Psd95*) (Supplementary Fig. 7d). By contrast, the inactive mutant failed to alter the expression of these genes (Supplementary Fig. 7d). Taken together, HMGCS2 upregulation restores the synaptic plasticity by mediating the expression of NMDARs and Syn1 via H3K9bhb.

**Fig. 9:**
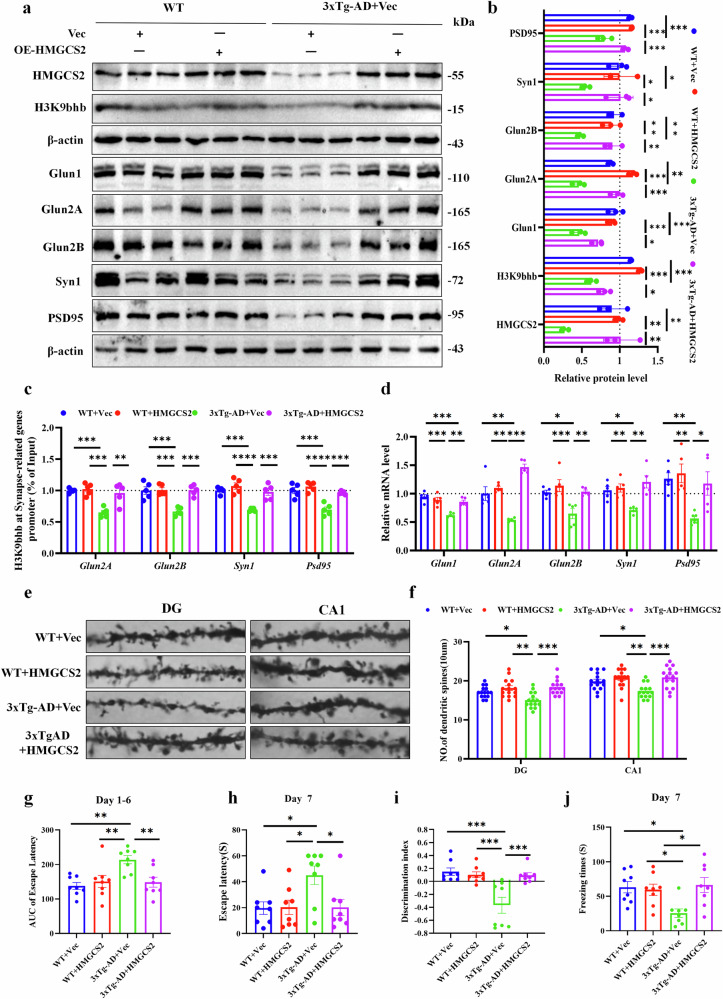
HMGCS2 overexpression improves cognitive function via H3K9bhb-mediated synaptic gene expression in 3xTg-AD mice. **a**,**b**, A western blot analysis (**a**) of hippocampal lysates shows that HMGCS2 upregulation increases the protein levels (**b**) of H3K9bhb, Glun1, Glun2A, Glun2B, Syn1 and PSD95, *n* = 3 per group. **c**, The ChIP–qPCR analysis of H3K9bhb enrichment at the promoters of *Glun2A*, *Glun2B*, *Syn1* and *PSD95* in the four groups, *n* = 5 per group. **d**, The mRNA levels of Glun1, Glun2A, Glun2B, Syn1 and PSD95 in the hippocampus, as determined by RT–qPCR, *n* = 5 per group. **e**,**f**, Golgi staining reveals increased dendritic spine density in 3xTg-AD mice following overexpression of HMGCS2; representative images (**e**) and quantification (**f**) are shown, *n* = 5 per group, three fields per mice. Scale bar, 5 μm. **g**–**j**, A behavioral assessment of spatial learning and memory using the MWM, NOR and contextual fear conditioning tests: area under the curve (AUC) of escape latency during MWM training of day 1–6 (**g**), escape latency on day 7 of the MWM test (**h**), NOR discrimination index (**i**), freezing time on day 7 in the contextual fear conditioning test (**j**), *n* = 8 per group. Data are shown as mean ± s.e.m. One-way ANOVA followed by Bonferroni’s post hoc test for **b**–**d**, **f** and **g**–**j**. ^*^P < 0.05, ^**^P < 0.01^, ***^P < 0.001, ^****^P < 0.0001; ns, not significant.

To investigate the critical role of HMGCS2 in ameliorating synaptic and cognitive deficits in AD via promoting β-OHB and H3K9bhb production, we injected 7-month-old 3xTg-AD mice and WT controls with HMGCS2-overexpressing or control viral vectors (Supplementary Fig. 8). Two months post-injection, western blot analysis revealed that HMGCS2 overexpression significantly increased the levels of H3K9bhb and key synaptic proteins (Glun1, Glun2A, Glun2B, Syn1 and PSD95) in the hippocampus of 3xTg-AD mice (Fig. 9a, b). Further qPCR and ChIP–qPCR analysis showed that compared with the 3xTg-AD+Vec group, synaptic related genes were significantly upregulated in the 3xTg-AD + HMGCS2 group, which was associated with a significant increase in H3K9bhb enrichment in the promoter region (Fig. 9c, d). Golgi staining demonstrated a marked reduction in dendritic spine density in DG and CA1 hippocampal neurons of 3xTg-AD mice relative to WT controls. HMGCS2 overexpression significantly increased total spine density, suggesting enhanced synaptic stability and structural plasticity (Fig. 9e, f). Sholl analysis indicated that 3xTg-AD mice exhibited shorter total dendritic length and reduced dendritic branching complexity. The HMGCS2 overexpression increased dendritic intersections, branch points and total length in DG and CA1 neurons (Supplementary Fig. 9a–g). Together, these findings demonstrate that the improvement in synaptic plasticity following HMGCS2 upregulation is mediated by H3K9bhb in 3xTg-AD mice.

Given the involvement of HMGCS2 in synaptic plasticity regulation via H3K9bhb in 3xTg-AD mice, we further investigated whether HMGCS2 overexpression could ameliorate cognitive deficits in this AD model. In the MWM test, 3xTg-AD mice exhibited significantly longer escape latencies during the training phase, whereas HMGCS2 overexpression substantially shortened the time required to locate the platform (Fig. 9g and Supplementary Fig. 10a). During the probe trial on day 7, 3xTg-AD mice again showed prolonged escape latencies, which were significantly reduced upon HMGCS2 overexpression (Fig. 9h and Supplementary Fig. 10b). Furthermore, 3xTg-AD mice crossed the platform location less frequently, an effect that was reversed by HMGCS2 overexpression (Supplementary Fig. 10b, c). Importantly, no significant difference in total distance traveled was observed among the groups (Supplementary Fig. 10d), ruling out potential confounds from locomotor activity on the cognitive behaviors assessed. In the NOR test, 3xTg-AD mice failed to display a preference for the novel object; however, HMGCS2 overexpression significantly increased the time spent exploring the novel object (Supplementary Fig. 10e). The discrimination index in 3xTg-AD mice was also significantly lower than that in the WT+Vec mice or 3xTg-AD + HMGCS2 mice (Fig. 9i). In the contextual fear conditioning test, 3xTg-AD mice exhibited significantly reduced freezing times on both day 1 and day 7 compared with WT controls, and this impairment was rescued by HMGCS2 overexpression (Fig. 9j and Supplementary Fig. 10f), indicating that HMGCS2 can not only reverse short-term memory but also improve long-term memory in 3xTg AD mice. Collectively, these results demonstrate that HMGCS2 overexpression significantly improves cognitive deficits in 3xTg-AD mice.

## Discussion

In the present study, we have found that β-OHB and H3K9bhb were reduced in the brain of 3xTg-AD mice and patients with AD. The 3xTg-AD mice exhibited a low enrichment of H3k9bhb in the promoters of axon guidance- and synapse-related genes, whereas β-OHB replenishment enhanced H3K9bhb level and its enrichment in the promoter regions of axon guidance- and synapse-related genes. In addition, HMGCS2 was decreased in the brain of AD mice and patients with AD, as well as Aβ-treated N2a cells and 3xTg-AD primary neurons, whereas upregulating HMGCS2 significantly increased the expression of H3K9bhb and its enrichment in the promoter regions of synapse-related genes. These findings document the involvement of HMGCS2 in histone β-hydroxybutyrylation to regulate the expression of synapse-related genes in the AD brain and further demonstrate the promising therapeutic potential of targeting HMGCS2 and β-OHB supplement in treating patients with intermediate or advanced AD.

Histone modification, as one of the types of epigenetic regulation, plays an important role in the regulation of cognitive function by affecting chromatin remodeling and participating in the regulation of many neuronal functions, from synaptic plasticity to learning and memory^12,13^. The ketone body β-OHB, produced via fatty acid β-oxidation under conditions such as fasting or ketogenic diet, enacts signaling by directly enhancing histone lysine β-hydroxybutyrylation to regulate gene expression and other histone modifications^30,32^. β-OHB is significantly reduced in the blood and brain tissues of patients with AD^21^, whereas β-OHB supplementation can enhance synaptic plasticity in AD mice^22^. In the current study, the hippocampal levels of β-OHB and H3K9bhb were significantly decreased in 9-month-old 3xTg-AD mice, whereas β-OHB treatment significantly increased the hippocampal levels of β-OHB, and H3K9bhb further improved the cognitive function of the mice. Therefore, β-OHB directly enhances H3K9bhb to participate in the regulation of cognitive function in AD, and beneficial interventions to improve β-OHB such as caloric restriction and ketogenic diet are essential to delay the progression of AD.

Studies have shown that β-OHB can slow down cognitive impairment in AD mice by improving synaptic plasticity^26^, but the specific mechanism is still unclear. Axon guidance is the necessary processes for the neural circuits formation^33^, and axon guidance molecules play an important role in guiding growth cones to form synapses and participate in cognitive damage caused by Aβ and tau pathology through various signaling pathways^34^. In addition, NMDA receptors regulate the production and maintenance of long-term potentiation and long-term depression, which are crucial for synaptic plasticity and learning and memory^31^. However, NMDARs are significantly impaired in AD^35^. Here, we found that the H3K9bhb enrichment in the axon guidance genes and NMDARs promoter region of middle-aged 3xTg AD mice was reduced, indicating that the loss of axon guidance genes and NMDAR transcription in AD may be caused by abnormal histone β-hydroxybutyrylation. Correspondingly, increasing H3K9bhb by supplementing β-OHB can restored the expression of axon guidance genes and NMDARs, ultimately improving synaptic plasticity and cognitive function.

β-OHB, as a direct donor of Kbhb modification, is the main component of ketones, which is generated through a series of metabolic reactions under the action of related enzymes, as follows: HMGCS2 catalyzes the condensation of AcAc-CoA and acetyl-CoA to form HMG-CoA, which is then cleaved into acetyl-CoA and AcAc by HMGCL, the latter ultimately generates OHB through the action of BDH1^19^. Among them, HMGCS2 and BDH1, as key enzymes in ketone metabolism, are closely related to the modification of histone Kbhb. The deficiency of HMGCS2 in the intestine can impair H3K9bhb aggregation and affect the metabolic gene program related to H3K9bhb^30^, whereas inhibiting BDH1 can lead to β-OHB aggregation increases H3K9bhb and promotes liver cancer cell proliferation^20^. Here, through AD data analysis and experimental verification, we have found that, among the enzymes related to β-OHB production, only HMGCS2 shows reduced consistency in patients with AD and 3xTg-AD mice brain tissues, as well as Aβ-treated N2a cells and 3xTg-AD primary neurons. However, the upregulation of HMGCS2 levels significantly increased β-OHB and H3K9bhb levels, thereby promoting synaptic related genes expression and synaptic plasticity. These results suggest that HMGCS2 may improve cognitive function in patients with AD by promoting the production of β-OHB and increasing H3K9bhb. Therefore, the development of agonists targeting HMGCS2 is expected to become one of the new approaches for AD treatment. Given that cognitive impairment in AD is directly related to synaptic function, interventions based on the ‘HMGCS2/β-OHB/H3K9bhb regulation of synaptic plasticity’ will provide a new perspective for the treatment of AD.

Of note, although our findings primarily focus on the epigenetic mechanism mediated by H3K9bhb, the therapeutic effects of β-OHB in AD are probably multifaceted. Beyond its role in histone modification, β-OHB, as a major component of ketone bodies, can serve as an alternative energy source to glucose in the brain. Given the impaired cerebral energy metabolism observed in AD, β-OHB enhances mitochondrial function, boosts ATP production and ameliorates neuronal energy deficits, thereby alleviating energy deprivation-induced neuronal damage^26,36,37^. In addition, β-OHB suppresses microglial overactivation and reduces the release of proinflammatory cytokines such as IL-1β and TNF-α, attenuating neuroinflammation^21,26,38^. Moreover, β-OHB may exert neuroprotection through receptor-mediated signaling pathways, such as via GPR109A activation, modulating amyloid precursor protein (APP) expression and upregulating neuroprotective proteins including neurofilament light chain (NEP)^26^. Collectively, these observations underscore the multifaceted neuroprotective role of β-OHB in AD. Our study delineates a pathway from β-OHB metabolism to H3K9bhb-mediated epigenetic regulation of synaptic plasticity, highlighting a promising therapeutic strategy for AD.

Although our study provides key insights into the role of H3K9bhb in gene regulation, several limitations should be noted. The CUT&Tag data, although informative, yielded weak peak intensities, potentially owing to either limited antibody efficiency in this application or low endogenous levels of H3K9bhb. Consequently, the CUT&Tag profiling data are semiquantitative and only serve as suggestive evidence. However, our central conclusions are robustly supported by orthogonal, quantitative ChIP–qPCR experiments that rigorously validated the H3K9bhb enrichment changes at specific promoter regions of interest.

**Fig. 10:**
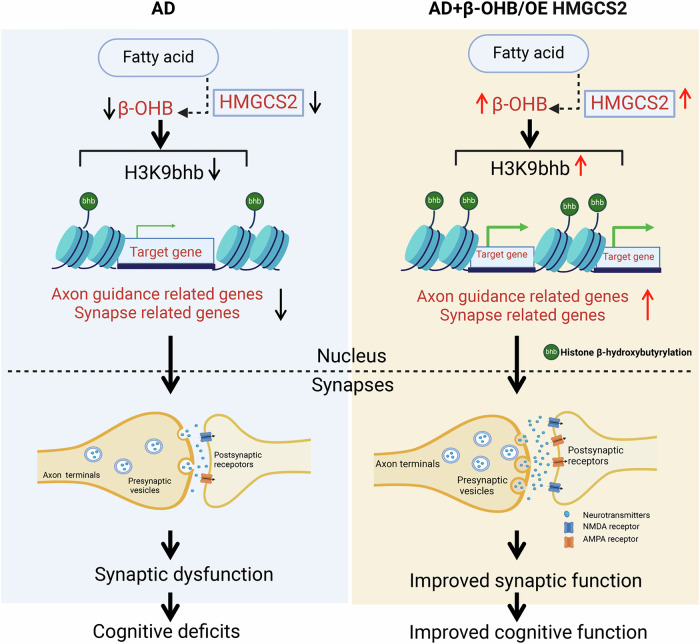
HMGCS2-dependent β-OHB/H3K9bhb ameliorates synaptic plasticity and cognition in Alzheimer’s disease. HMGCS2 deficiency in AD reduces β-OHB-mediated H3K9bhb, suppressing the transcription of synaptic genes and leading to cognitive decline. Conversely, increasing HMGCS2 expression or β-OHB levels promotes H3K9bhb-dependent synaptic genes expression, restoring synaptic integrity and improving cognitive function. Abbreviations: HMGCS2, 3-hydroxy-3-methylglutaryl-CoA synthase 2; β-OHB, β-hydroxybutyrate; H3K9bhb, histone H3 lysine 9 β-hydroxybutyrylation; OE, overexpression.

Recent studies have found that histone modification, including histone acetylation (H3K27, H3K9, H4K12)^39,40^, methylation (H3K9)^41^ and lactylation (H4K12)^23^, plays an important role in AD-like neurodegeneration and cognitive impairment. However, as a new modification, histone β-hydroxybutyrylation has not been reported in AD. Here, we have revealed that HMGCS2 was a key molecule in cognitive impairment and that targeting HMGCS2 or supplementation with β-OHB improves synaptic plasticity and cognitive function via increasing H3K9bhb (Fig. 10). In sum, our results have identified an epigenetic mechanism ‘HMGCS2/β-OHB/H3K9bhb’ in AD and provide a new strategy and target for the treatment of intermediate or advanced AD.

## Supplementary information

Supplementary Information

## Data Availability

All data used to support the findings of this study are included within the Article, and raw data are available from the corresponding author.
