# Supplementary material for: HMGCS2-dependent β-OHB/H3K9bhb ameliorates synaptic plasticity and cognition in Alzheimer’s disease
Source: Exp Mol Med. 2026 Mar 6;58(3):813–31. doi: 10.1038/s12276-026-01664-9 (PMC13049083; doi:10.1038/s12276-026-01664-9)
Supplement: Supplementary file 1 — Supplementary Information [file 12276_2026_1664_MOESM1_ESM.pdf]

## Supplementary Figure legends

**Supplementary Fig 1. β-OHB supplementation increases H3K9bhb level in the hippocampus of 3xTg-AD mice.**

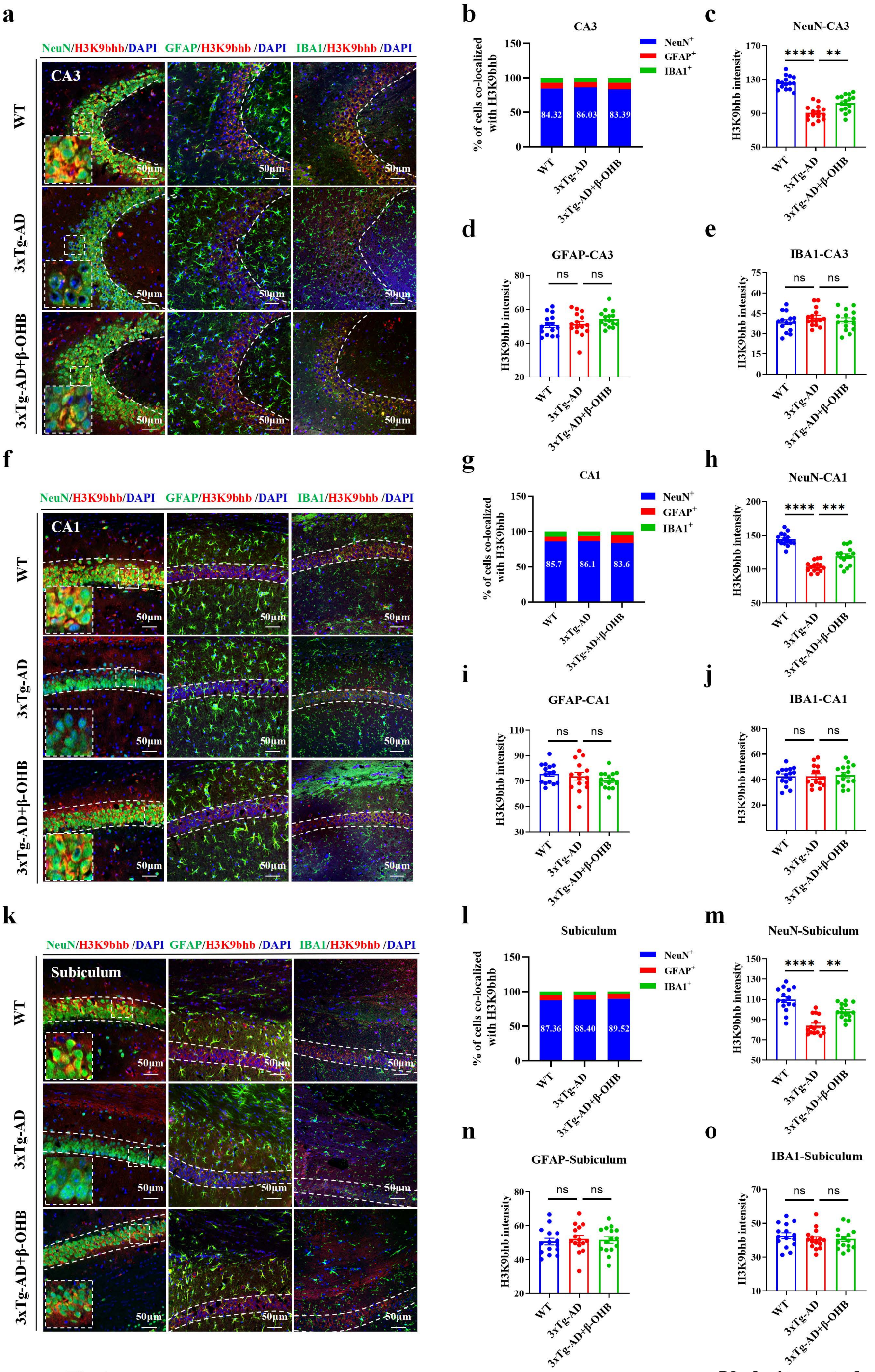


Supplementary Fig 1. Yu haitao et al., 2025

**Supplementary Fig. 1 β-OHB supplementation increases H3K9bhb level in the hippocampus of 3xTg-AD mice.** (a-e) Representative images (a), % of cells co-localized with H3K9bhb quantitative analysis (b) and H3K9bhb fluorescence in hippocampal CA3 region neurons and glial cells across three experimental groups (c-

e), $n$=15 cells. Scale bar, 50 μm. (f-j) Representative images (f), % of cells co-localized with H3K9bhb quantitative analysis (g) and H3K9bhb fluorescence in hippocampal CA1 region neurons and glial cells across three experimental groups (h-j), $n$=15 cells. Scale bar, 50 μm. (k-o) Representative images (k), % of cells co-localized with H3K9bhb quantitative analysis (l) and H3K9bhb fluorescence in hippocampal CA1 region neurons and glial cells across three experimental groups (m-o), $n$=15 cells. Scale bar, 50 μm. Data were shown as mean ± SEM. One-way ANOVA followed by Bonferroni's post hoc test for C, D, E, H, I, J, M, N, O. $^{*}P$ <0.05, $^{**}P$ < 0.01, $^{***}P$ < 0.001, $^{****}P$ < 0.0001; ns., not significant.

**Supplementary Fig 2. Transcriptomes revealed that β-OHB supplementation improved axon guidance function in 3xTg-AD mice.**

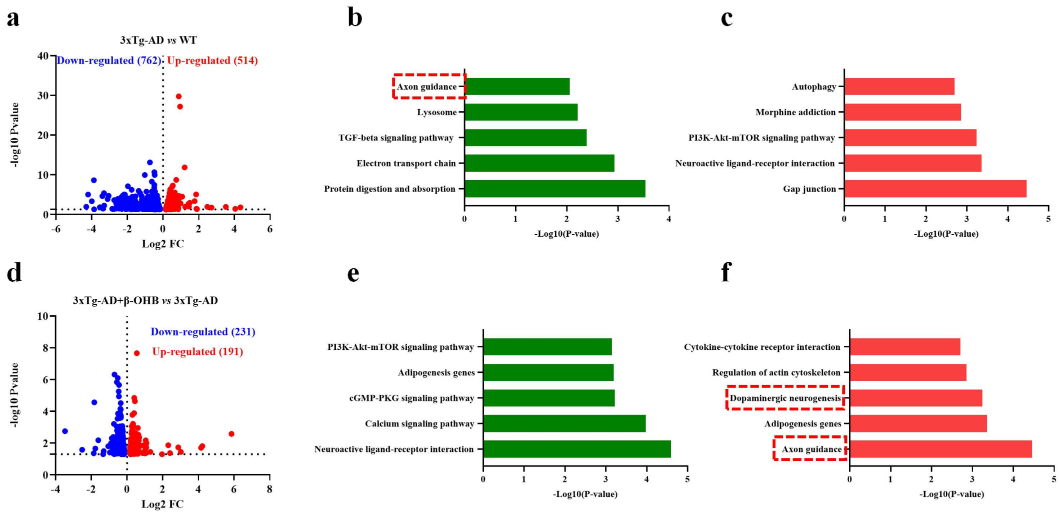


**Supplementary Fig 2.** **Yu haitao et al., 2025**

**Supplementary Fig. 2 Transcriptomes revealed that β-OHB supplementation improved axon guidance function in 3xTg-AD mice.** (a and d) The volcano plot shows significantly differentially expressed genes (p-value<0.05, Ratio>1.1), with red indicating upregulation and blue indicating downregulation, $n$=3/group. (b and e) The KEGG pathway was enriched by down-regulated genes. (c and f) The KEGG pathway was enriched by up-regulated genes, $n$=3/group.

**Supplementary Fig 3. Repeated H3K9bhb CUT&Tag experiment: β-OHB replenishment enhanced H3K9bhb enrichment in the promoter regions of related genes.**

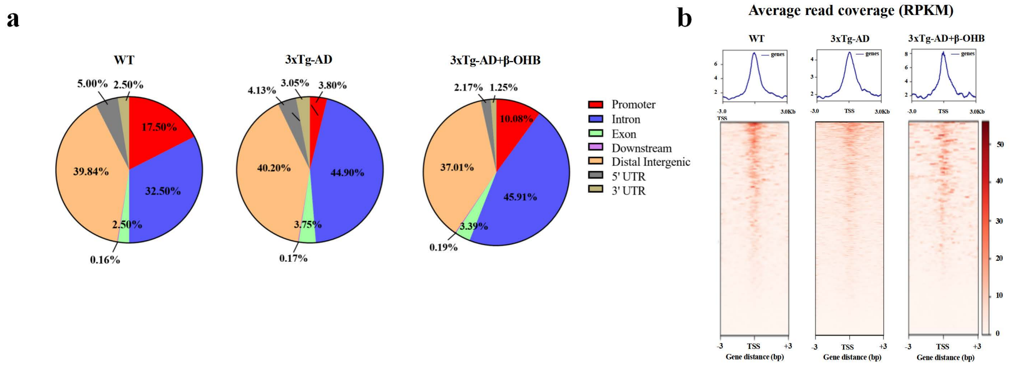


Supplementary Fig 3. Yu haitao et al., 2025

**Supplementary Fig. 3 Repeated H3K9bhb CUT&Tag experiment: β-OHB replenishment enhanced H3K9bhb enrichment in the promoter regions of related genes.** (a) Genome-wide distribution of H3K9bhb-binding peaks in the hippocampus of from WT, 3xTg-AD and 3xTg-AD+β-OHB mice. (b) The binding density of H3K9bhb was visualized by deepTools: the heatmap presents the CUT&Tag tag counts on the different H3K9bhb binding peaks in hippocampus extracts between WT, 3xTg-AD and 3xTg-AD+β-OHB mice, ordered by signal strength.

**Supplementary Fig 4. β-OHB supplementation can effectively improve hippocampal synaptic function in 3xTg-AD mice**

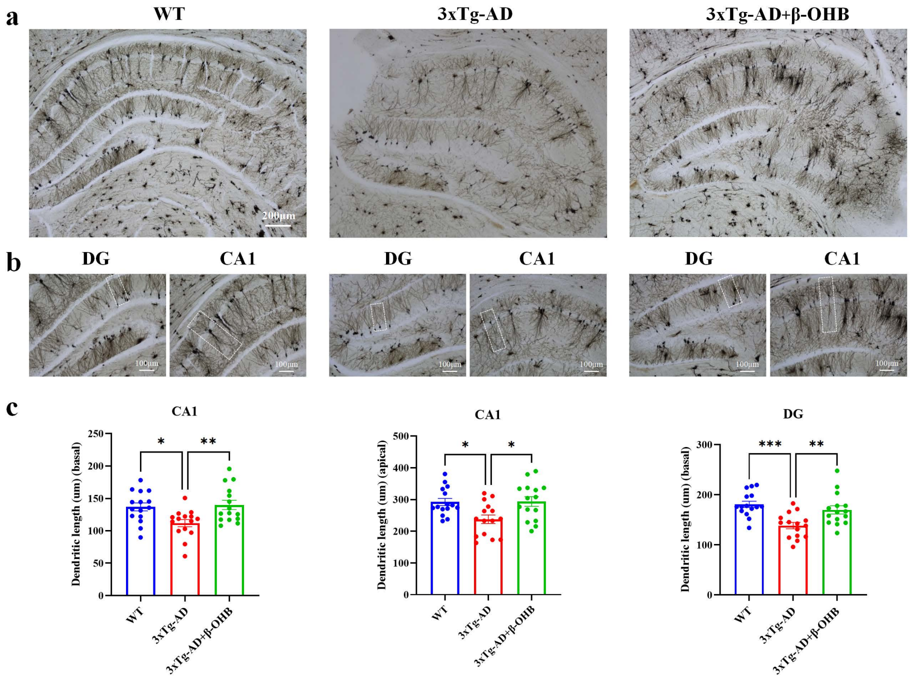


Supplementary Fig 4. Yu haitao et al., 2025

**Supplementary Fig. 4 β-OHB supplementation can effectively improve hippocampal synaptic function in 3xTg-AD mice** (a-c) Sholl analysis showed synaptic complexity of neurons after supplementing with β-OHB in 3xTg-AD mice. The representative images (a and b) and the quantitative analysis, *n*=5/group, 3 fields/mice (c). Data were shown as mean ± SEM. One-way ANOVA followed by Bonferroni's post hoc test for C. $^{*}P$ <0.05, $^{**}P < 0.01$, $^{***}P < 0.001$, $^{****}P < 0.0001$.

**Supplementary Fig 5.** **HMGCS2 was reduced in the brain tissues of 3xTg-AD mice and N2a-A$\beta_{1-42}$ cells.**

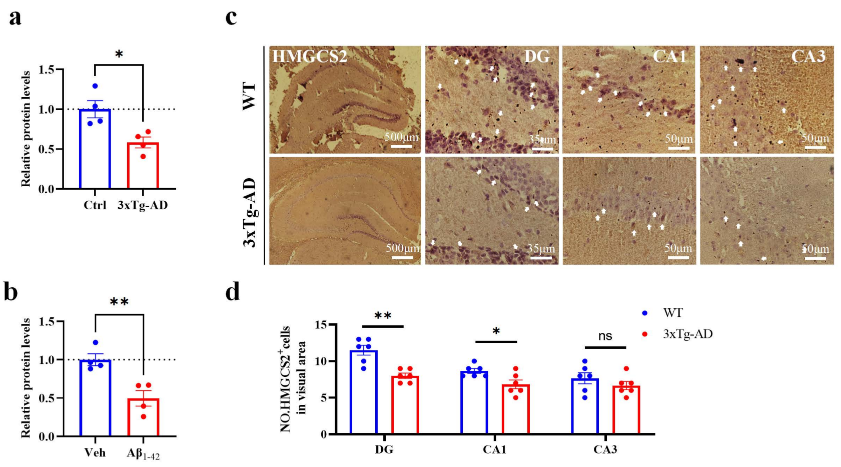


Supplementary Fig 5. Yu haitao et al., 2025

**Supplementary Fig. 5 HMGCS2 was reduced in the brain tissues of 3xTg-AD mice and N2a-A$\beta_{1-42}$ cells.** (a-b) Quantitative analysis of HMGCS2 protein level in primary cells from 3xTg-AD mice, *n*=4/group (a) and N2a-A$\beta_{1-42}$ cells, *n*=4/group (b). (c and d) Immunohistochemistry staining revealed a decrease in HMGCS2 levels in the hippocampal subsets of 3xTg-AD mice in comparison to the control group, *n*=5/group, 2 fields/mice. Scale bar, 50 μm. Data were shown as mean ± SEM. Unpaired t-test for A, B, D. $^{*}P$ <0.05, $^{**}P < 0.01$, $^{***}P < 0.001$, $^{****}P < 0.0001$.

**Supplementary Fig 6. PPARα is the transcription factor of HMGCS2 in the hippocampus of 3xTg AD mice.**

**a**

| Matrix ID | Name | Score | predicted sequence |
|---|---|---|---|
| MA1148.2. | PPARα | 14.894 | AACTGGGTCAAAGGGCT |
| MA1947.2 | FOXO1 | 14.780 | ATAAACAGGA |
| MA0162.2 | EGR1 | 12.043 | TAGCCTCCCACTCC |
| MA0102.5 | CEBPA | 9.948 | GTTGCAGAAT |
| MA1533.2 | NR1I2 | 9.3788 | TGAGCCCTTTGACCC |

**b**

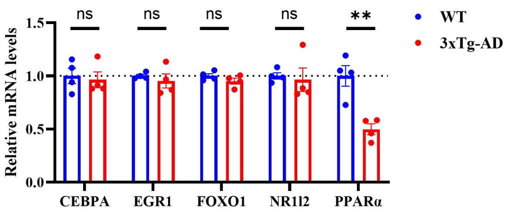


Supplementary Fig 6.

Yu haitao et al., 2025

**Supplementary Fig. 6 PPARα is the transcription factor of HMGCS2 in the hippocampus of 3xTg AD mice.** (a) Five transcription factors of HMGCS2 predicted by Jaspar. (b) mRNA expression levels of the five transcription factors in 3xTg-AD, n=4/group.Data were shown as mean ± SEM. Unpaired t-test for B. $^{*}P$ <0.05, $^{**}P$ < 0.01, $^{***}P$ < 0.001, $^{****}P$ < 0.0001.

**Supplementary Fig 7. Mutation of HMGCS2 did not rescue H3K9bhb levels or synaptic gene transcription in primary hippocampal neurons of 3xTg-AD mice.**

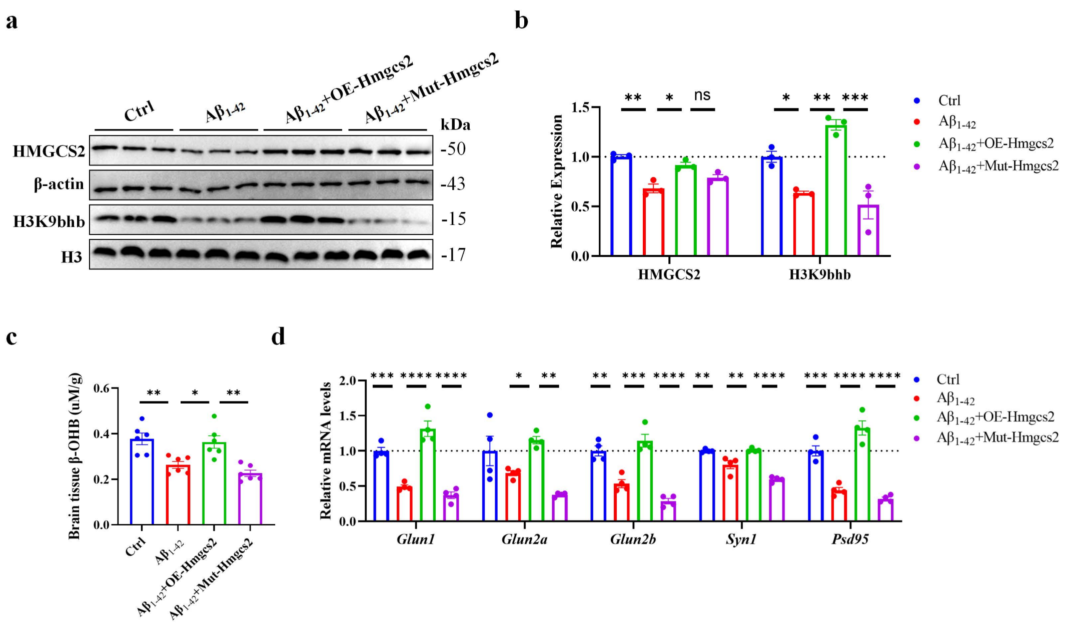


Supplementary Fig 7. Yu haitao et al., 2025

**Supplementary Fig. 7 Mutation of HMGCS2 did not rescue H3K9bhb levels or synaptic gene transcription in primary hippocampal neurons of 3xTg-AD mice.** (a-b) Western blot analysis (a) and quantitative data (b) of H3K9bhb levels after mutation of the HMGCS2 gene, n=3/group. (c) β-OHB levels measured by ELISA after mutation of the HMGCS2 gene, n=6/group. (d) mRNA expression levels of synaptic genes after mutation of the HMGCS2 gene, n=4/group. Mut= Mutation. Data were shown as mean ± SEM. One-way ANOVA followed by Bonferroni's post hoc test for B, C, D. $^{*}P$ <0.05, $^{**}P < 0.01$, $^{***}P < 0.001$, $^{****}P < 0.0001$.

Supplementary Fig 8. Schematic diagram of HMGCS2 overexpression virus.

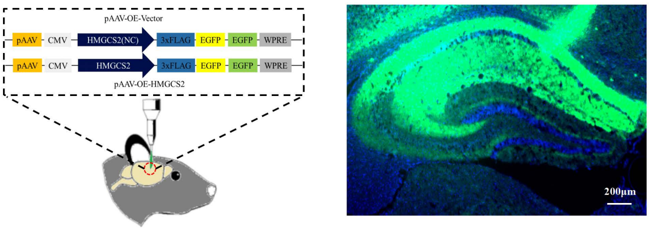


Supplementary Fig 8. Yu haitao et al., 2025

**Supplementary Fig. 8 Schematic diagram of HMGCS2 overexpression virus.**

Supplementary Fig 9. HMGCS2 overexpression improves hippocampal synaptic function in 3xTg-AD mice.

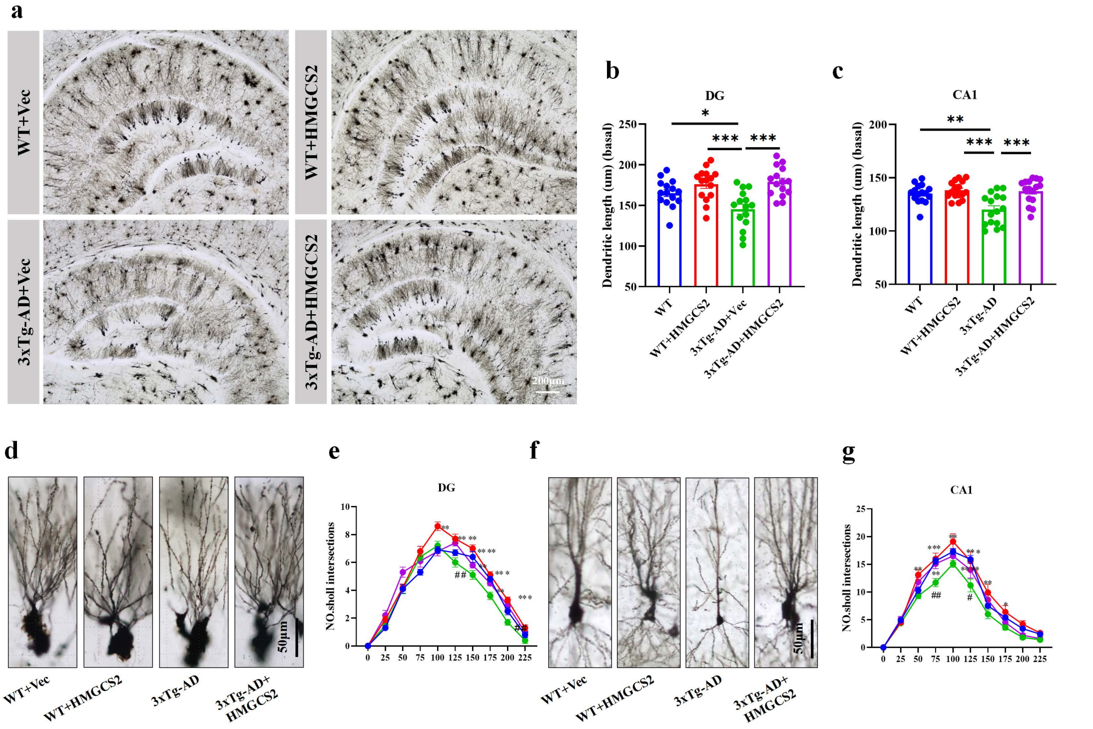


Supplementary Fig 9. Yu haitao et al., 2025

**Supplementary Fig. 9 HMGCS2 overexpression improves hippocampal synaptic function in 3xTg-AD mice.** (a–c) Golgi staining shows increased dendritic branch

length following HMGCS2 overexpression in 3xTg-AD mice. Representative image (a) and quantification (b, c). Scale bar, 200 μm, *n*=5/group, 3 fields/mice. (d–g) Sholl analysis reveals enhanced dendritic complexity in neurons from 3xTg-AD mice after HMGCS2 overexpression. Representative diagrams, Scale bar, 50 μm (d, f) and quantitative results (e, g), *n*=5/group, 3 fields/mice. Data were shown as mean ± SEM. One-way ANOVA followed by Bonferroni's post hoc test for B, C. Two-way ANOVA followed by Bonferroni's post hoc test for E, G. $^{*}P$ <0.05, $^{**}P$ < 0.01, $^{***}P$ < 0.001, $^{****}P$ < 0.0001.

**Supplementary Fig 10. HMGCS2 overexpression ameliorates learning and memory in 3xTg-AD mice.**

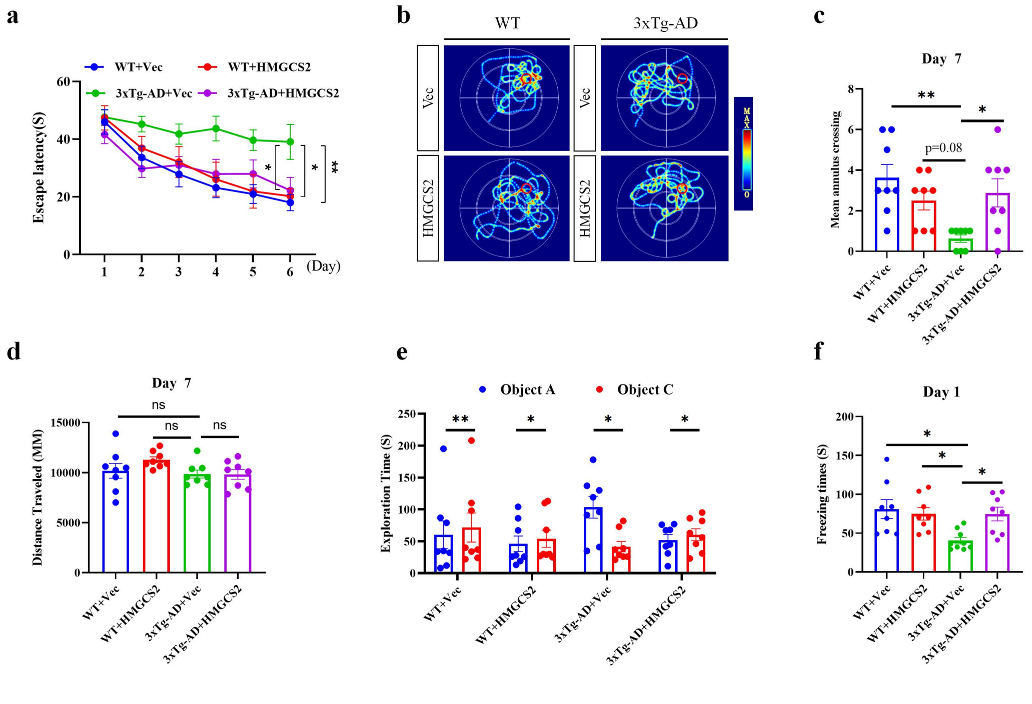


Supplementary Fig 10. Yu haitao et al., 2025

**Supplementary Fig. 10 HMGCS2 overexpression ameliorates learning and memory in 3xTg-AD mice.** (a) Escape latency during training in the Morris water maze test. (b) Representative swimming paths to the target platform on day 7. (c) Number of platform crossings in the target quadrant on day 7. (d) Swimming distance of mice. (e) Exploration time for a novel object on days 1 and 2 in the novel object recognition test. (f) Freezing time on day 1 in the contextual fear conditioning test. *n*=8/group. Data were shown as mean ± SEM. Unpaired t-test for E, One-way

ANOVA followed by Bonferroni's post hoc test for C, D, F. Two-way ANOVA followed by Bonferroni's post hoc test for A. $^{*}P$ <0.05, $^{**}P$ < 0.01, $^{***}P$ < 0.001, $^{****}P$ < 0.0001.

**Supplementary Table. 1 Information of human brain sections.**

| | Type | Dementia degree | Age | Gender | PMI(h) | Cause of death | Braak Stage |
|---|---|---|---|---|---|---|---|
| 1 | HC | - | 25 | Female | 12 | Fall to death | - |
| 2 | HC | - | 62 | Female | 84 | Carotid artery rupture | - |
| 3 | HC | - | 48 | Male | 96 | Organophosphorus | - |
| 4 | HC | - | 52 | Male | 120 | Fall to death | - |
| 5 | AD | +++ | 74 | Male | 12 | Acute myocardial ischemia | III |
| 6 | AD | ++ | 53 | Male | 48 | Hypertension | III |
| 7 | AD | ++ | 62 | Female | 72 | Septicopyemia | II |
| 8 | AD | + | 58 | Male | 144 | Hemorrhagic shock | II |

AD = Alzheimer's desiease; HC = Health Control; PMI = Post Mortem Interval.
